# Changing sea ice cover led to marine biodiversity shifts in the Late Quaternary

**DOI:** 10.1038/s43247-026-03696-5

**Published:** 2026-06-04

**Authors:** Stijn De Schepper, Tristan Cordier, Katrine Sandnes Skaar, Jessica Louise Ray, Henrik Sadatzki, Bjørg Risebrobakken

**Affiliations:** 1 University of Bergen, Bjerknes Centre for Climate Research, Bergen, Norway; 2 NORCE Research, Bjerknes Centre for Climate Research, Bergen, Norway; 3 NORCE Research, Bergen, Norway; 4 Alfred Wegener Institute Helmholtz Centre for Polar and Marine Research, Bremerhaven, Germany; 5 Present Address: Aqua kompetanse AS, Flatanger, Norway

**Keywords:** Palaeoceanography, Palaeoclimate, Environmental impact, Microbiology

## Abstract

Today, the sea ice cover is rapidly declining in the Arctic Ocean. While major changes in the Arctic marine ecosystems are starting to appear, the full implication of a changing Arctic ecosystem is not well understood. Here we show, using sedimentary ancient DNA metabarcoding to document marine eukaryotic communities, that higher diversity is recorded in a warmer, sea ice free Labrador Sea compared to when this region is sea ice covered. Changes in diversity and communities happen slower than the rapid climate warming of Greenland Interstadial 8 (ca. 38,000 years ago) and the Bølling period (ca. 14,700 years ago). Surface ocean conditions, rather than atmospheric warming, exert control on the marine eukaryote community and diversity. Our observations from the paleoclimate record suggest that when sea ice further decreases under global warming, the (sub-)polar marine ecosystems will change drastically, and feedbacks to the carbon cycle and climate system become very likely.

## Abstract (web summary)

Changes to the sea ice cover in the Labrador Sea led to shifts in marine eukaryote communities, according to an analysis of ancient DNA in marine sediments from the past 50,000 years.

## Introduction

**Fig. 1:**
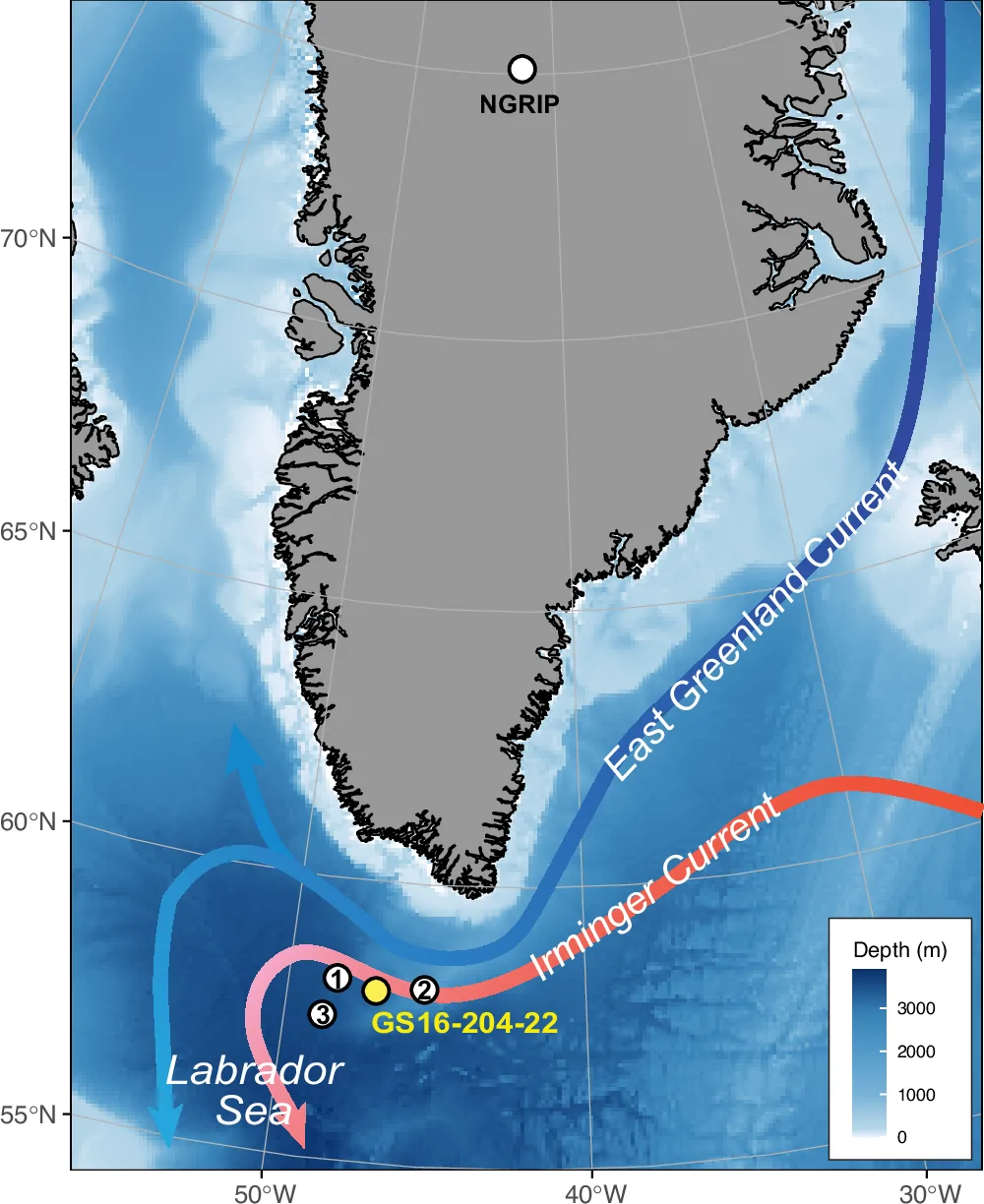
Study area. Location of the study site GS16-204-22 in the Labrador Sea, and nearby sites used in this study: 1 = HU90-13-13, 2 = GS16-204-23, 3 = MSM12/2-5-1.

In the coming decades, the Arctic is projected to transform from a sea ice covered, “white” ocean to a “blue”, summer sea ice free ocean^1–3^. The rapid reduction in sea ice extent and multi-year sea ice are already making a major imprint on Arctic Ocean biodiversity today^4–6^. While it is clear that changes to the Arctic ecosystem will continue as sea ice decreases^7,8^, the rate, magnitude and impact of this change to the ecosystem are unclear. As a case study for future Arctic change, we have turned to the geological record of the Labrador Sea and investigated how a changing sea ice environment impacted marine eukaryote communities over the past ca. 50,000 years. The oceanographic and climatic setting of the Labrador Sea and Arctic Ocean are different, but both rapid climate warming during the last glacial and long-term warming during the last deglaciation turned the sea-ice covered, “white” Labrador Sea into a seasonally sea-ice covered and eventually also “blue” sea. Indeed today, the Labrador Sea south of Greenland is sea ice free and influenced by warm Atlantic water from the Irminger Current and cool, polar water from the East Greenland Current (Fig. 1). During the last glacial (ca. 50,000 to 20,000 years ago), this region was covered by sea ice. Stable, multi-year sea ice conditions occurred during cold Greenland stadials (GS), whereas the warmer Greenland interstadials (GI) had unstable, seasonally sea ice free conditions^9–11^. On Greenland, temperature increased by 5 to 16 °C within decades during stadial to interstadial transitions^12^, leading to the replacement of the permanent sea ice cover in the Labrador Sea by seasonal sea ice to open water conditions^13^. These rapid changes to the sea ice environment of the Labrador Sea during warming events thus provide a unique window into the ecosystem response, a response that we may also see in the future Arctic.

While the mechanisms underlying rapid climate change events in the glacial ocean have been investigated, there is little known about how rapid sea ice change impacts marine biodiversity. Traditionally, paleoceanographic studies use microfossils, as these are widespread and often abundantly present in marine sediments. However, in sea-ice-covered oceans, calcareous and siliceous microfossils are often poorly preserved and show low diversity^14^. Marine sedimentary ancient DNA (*sed*aDNA) can provide a more complete picture of past biodiversity as it detects a wide range of planktonic and benthic organisms such as foraminifers, diatoms, dinoflagellates, chlorophytes, metazoan, cercozoan, copepods and several other benthic or planktic organisms^15–18^.

To document the effect of climate change and a changing sea ice environment on the ocean biosphere, we used metabarcoding to get a comprehensive view of eukaryotic biodiversity. Our study used sediments recording the last ca. 50,000 years of climate history in the Labrador Sea (GS16-204-22CC-B, 58° 02.83 N, 47° 02.36 W, water depth 3160 m; Fig. 1), including calving events from the Laurentide and Greenland Ice Sheets (Heinrich events) as well as rapid changes to the surface ocean and sea ice cover^9^. We extracted DNA from 114 sediment samples, with the highest temporal resolution around key rapid climate transitions. This was followed by PCR-amplification and sequencing of the V7 hypervariable region of the eukaryotic small subunit ribosomal RNA (18S SSU rRNA) on an Illumina MiSeq platform (*full details, see methods*). Our *sed*aDNA metabarcoding data shows that marine diversity and communities change when the sea ice cover changes and disappears.

## Results

We obtained 22,018,189 reads, which grouped into 4836 Amplicon Sequence Variants (ASV), from our 114 samples that cover the time interval between ca. 41.8 and 6.1 ka (all ages are reported in ka b2k, i.e. thousand years before 2000 AD; *see methods*). The ASVs belong to fossilising protists such as diatoms and dinoflagellates, but also to taxa that do not leave behind fossil remains such as cercozoans, apicomplexans or fungi. Our metabarcode data obtained from the sediment includes both benthic and planktic organisms, which we consider both to respond to changing sea ice conditions. This assumption is strengthened by the observation of a strong pelagic-benthic coupling in sea ice environments of the Labrador Sea^19^ or Arctic Ocean^20,21^. Also, it is important to note that our metabarcoding dataset, like any high-throughput sequencing dataset, is compositional^22,23^, meaning that the microbial load in the sample is unknown, and so the read counts of a given taxa is tightly dependent on all other taxa of the dataset. We therefore document community shifts in terms of changes in relative abundances in our data. A final consideration one must make when working with ancient DNA, is the possibility that shifts in marine communities may stem from DNA preservation problems. However, we find this unlikely and argue that the changes in communities presented here are not (strongly) impacted by DNA preservation. An earlier metabarcoding study showed community shifts in the considerably older sediments of the Last Interglacial from the same core^24^.

**Fig. 2:**
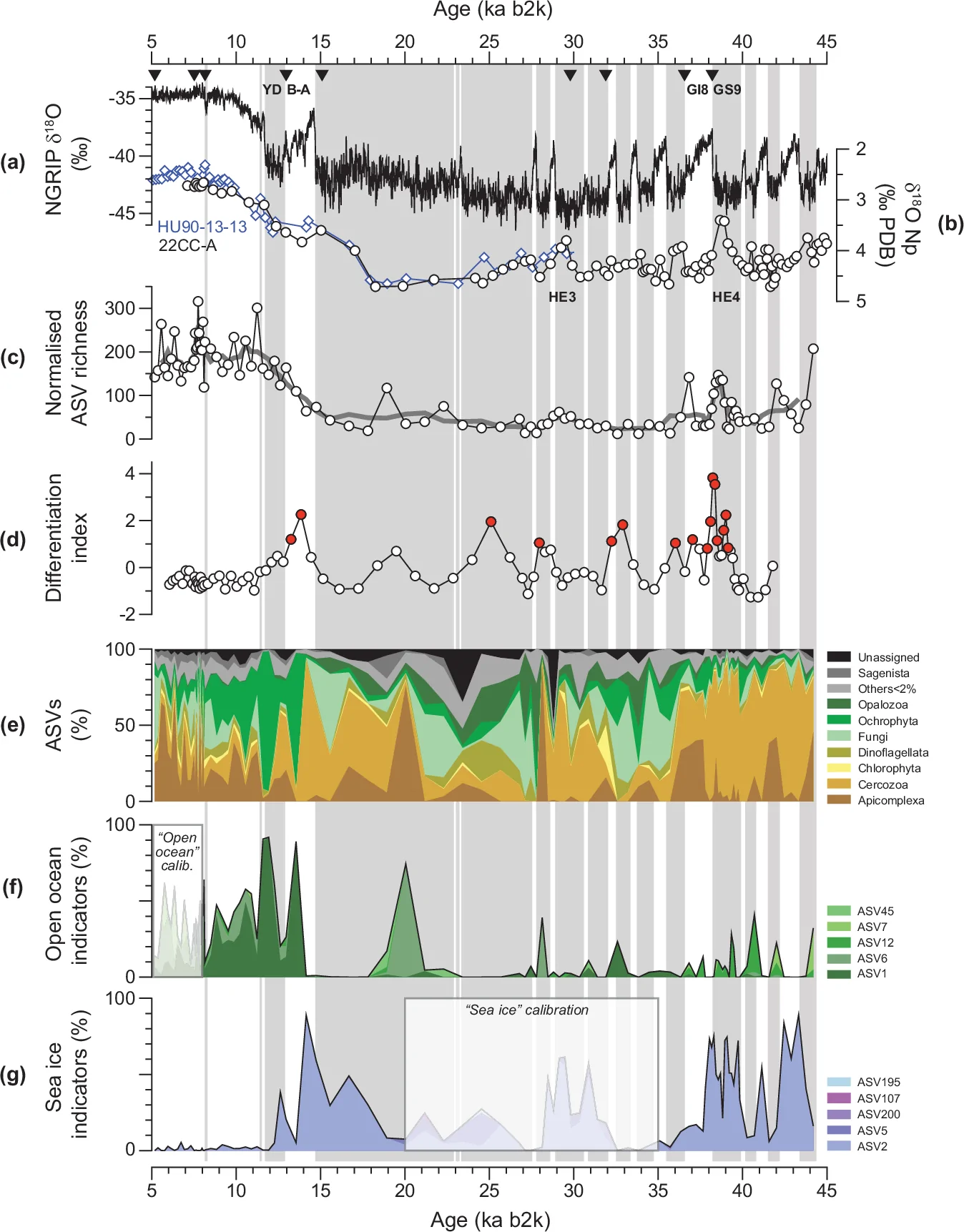
Paleoclimate, paleoceanography and marine community records for the last ca. 45,000 years in the Labrador Sea. **a** NGRIP oxygen isotope of water record^30^. Black triangles indicate age model tie points for 22CC-B (see Suppl. Table S2). **b** Planktic foraminifer *Neogloboquadrina pachyderma* oxygen isotopes of 22CC-A^9^, this study (black) and HU90-13-13^54^ (blue). **c** Normalised ASV richness, with 5-point running average (thick grey line). **d** Differentiation index of community composition (i.e. how the Bray-Curtis dissimilarity changes between pair of samples along the record), with statistically significant changes in composition shown as red dots. **e** Relative abundance of dominant high-rank taxonomic groups in 22CC-B as determined by metabarcoding. **f** Open ocean ASV indicators, including the calibration window. **g** Sea ice ASV indicators, including the calibration window. Background grey shading shows stadial-interstadial stratigraphy^55^. All plots show thousand years before 2000 AD (ka b2k). Abbreviations used: GS Greenland Stadials, GI Greenland Interstadials, YD Younger Dryas, B-A Bølling-Allerød, HE Heinrich Event.

Over the entire studied interval, we found eukaryotic communities (Fig. 2e) to be dominated by cercozoans (35% of the reads), apicomplexans (20%) and diatoms (15%). Richness (Fig. 2c) and relative abundances (Fig. 2e) of eukaryotic groups shifted throughout the record. Cercozoans dominated during 44–41 ka, 32–29 ka, and 19–15 ka, while fungi, alveolates and opalozoan dominated during 36–32 ka and 29–21 ka. We recorded substantial community shifts that are clearly expressed in the alpha diversity patterns (Fig. 2d) around Heinrich Event 4 (HE4) and following the onset of the Bølling-Allerød (B-A; 14.7–12.9 ka). The analysis of beta-diversity variation, using a split moving-window analysis of ecological differentiation^25^ (see Methods) along the sediment core, also revealed significant shifts in eukaryote communities (Fig. 2d). Due to a lower sampling resolution in parts of the record as well as site-specific sedimentation processes and rates^9^, not all Dansgaard-Oeschger cycles were recorded in the sediment or sampled by us. Our sampling was highly resolved for GS9–GI8 and the deglaciation and the high differentiation index values (with associated *p* < 0.05) indicated important community shifts around HE4 and the onset of GI8, as well as in the B-A. The significant community shifts around 33 ka, 27 ka and 25 ka could not be unequivocally tied to rapid climate events.

Our results show that shifts in marine diversity occurred during climate transition over the last 50 ka. Alpha diversity (Fig. 2c) was generally low in the glacial Labrador Sea, which is known as a low-productivity, multi-year sea-ice-covered ocean^9,13^. Rapid climate warming events during the glacial led to milder interstadials with seasonal sea ice^9,13^, suggesting that diversity could be higher. High diversity was recorded in single samples older than 42 ka, but given the limitations to the age model in this part of the record, the high diversity samples could not be assigned unquestionably to a stadial or interstadial. In GS9 and GI8 we record high diversity in several samples, when melting icebergs released ice rafted detritus (IRD) around HE4 and subsequently around the onset of GI8 when the temperature on Greenland increases by >4 °C within a few decades (Fig. 2a, c). The highest diversity of our studied interval was recorded during the Holocene, when the Labrador Sea south of Greenland was almost entirely sea ice-free^26^, following a steady increase during the last deglaciation. Below we discuss both climate transitions and community shifts in more detail.

**Fig. 3:**
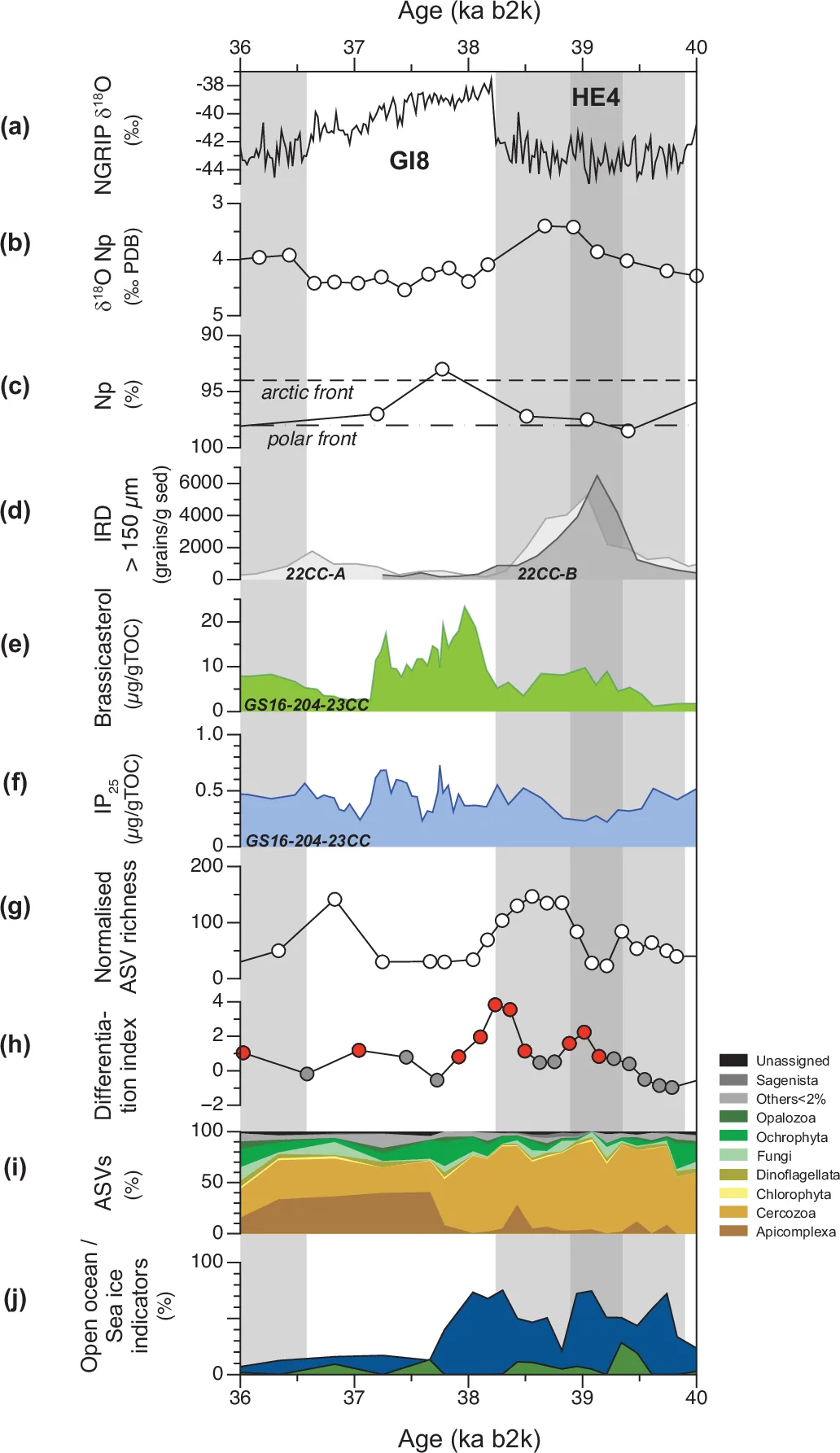
Paleoclimate, paleoceanography and marine community records from GS9 to GI8. **a** NGRIP oxygen isotope of water record^30^. **b** Planktic foraminifer *Neogloboquadrina pachyderma* oxygen isotopes of 22CC-A^9^ indicating fresher water around 38.5–39 ka. **c** Relative abundance of *N. pachyderma* in 22CC-A^9^. Values ≥ 98% indicate environments north of the Polar Front; values < 94% indicate environments south of the Arctic Front. **d** Ice rafted debris (IRD) records of 22CC-A^9^ (light grey) and 22CC-B (dark grey, *this study*). **e** Brassicasterol biomarker from GS16-204-23CC indicating overall marine productivity^13^. **f** Sea ice biomarker from GS16-204-23CC^13^. **g** Normalised ASV richness, with 5-point running average. **h** Differentiation index of community composition, which shows statistically significant changes in composition as red dots. **i** Relative abundance of dominant high-rank taxonomic groups in 22CC-B as determined by metabarcoding. **j** Open ocean and sea ice indicator ASVs. Background grey shading shows stadial-interstadial stratigraphy^55^. All plots show thousand years before 2000 AD (ka b2k).

Our results also show that the marine communities (Fig. 3i) were different in GS9 and GI8. Cercozoans dominated the assemblage with 79% of the reads during GS9 (39.7–39.3 ka). In the late part of GS9 (39.2–38.9 ka), at the peak IRD values of HE4, Cercozoans remained dominant (79%) but a major shift occurred in the Cercozoan composition: the relative abundance of Cryomonadida increased to 71% (from 58%) and Ebriida decreased to 4% (from 17%). Subsequently, in the latest part of GS9 (38.8–38.3 ka), we recorded a 10% decrease in Cercozoan relative abundance while the relative abundance of the apicomplexans increased to 10%, and diatoms to 5%. During the earliest part of GI8 (38.2–37.8 ka), Cercozoa remained dominant (64%), while diatoms reached on average 17% and maximally 24% of the assemblage. After 37.7 ka, the Apicomplexa (40%) became more abundant compared to the Cercozoa (27%).

**Fig. 4:**
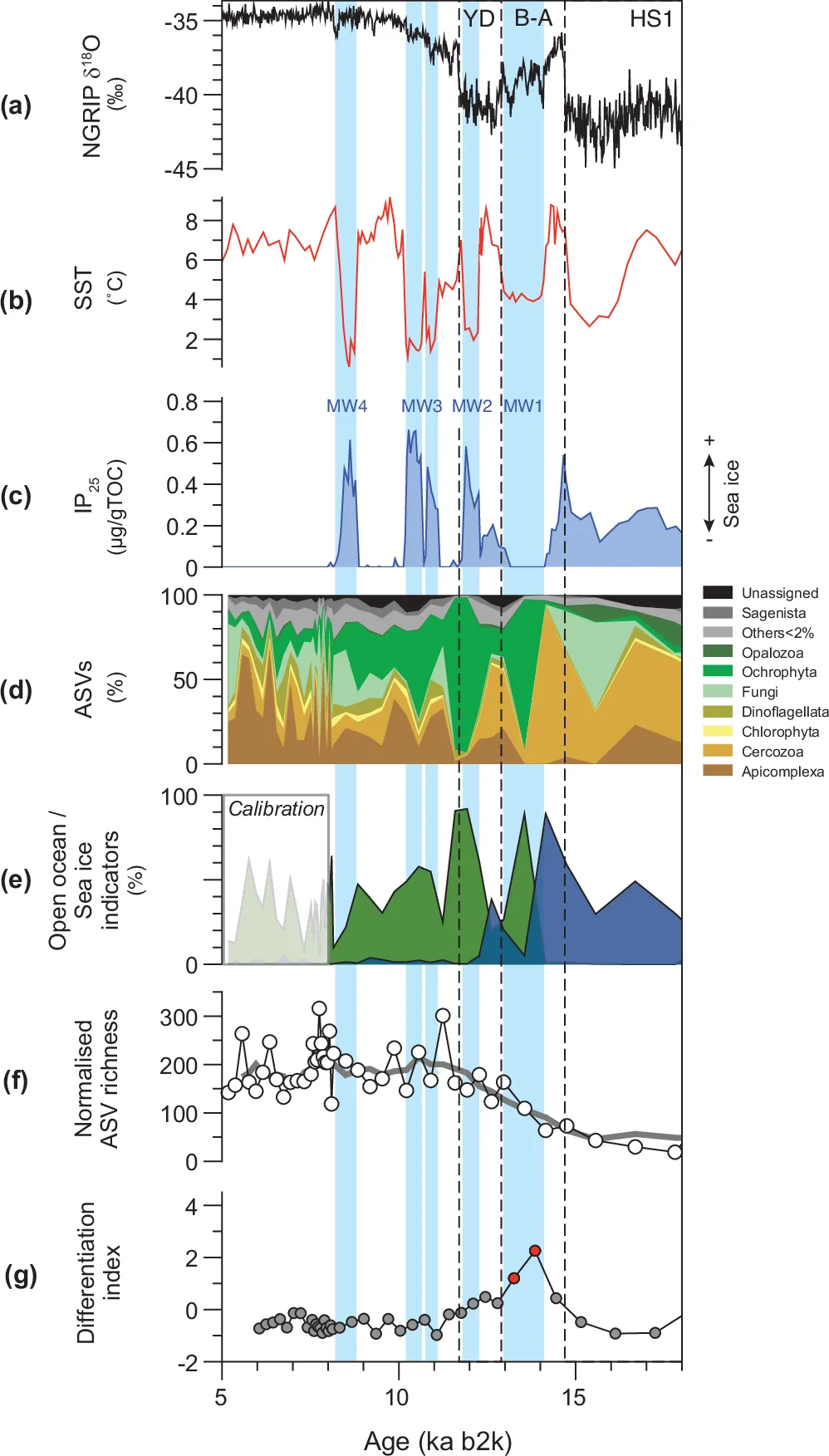
Paleoclimate, paleoceanography and marine community records for the deglaciation and Holocene, 18–5 ka. **a** NGRIP oxygen isotope of water record^30^. **b** Alkenone sea surface temperatures from MSM12/2-5-1^33^. **c** Sea ice biomarker IP_25_ from MSM12/2-5-1^33^. The IP_25_ pattern essentially mimics the PIP index^33^, supporting the interpretation of more (less) sea ice with higher (lower) values as indicated by the arrow. **d** Relative abundance of dominant high-rank taxonomic groups in 22CC-B as determined by metabarcoding. **e** Open ocean and sea ice indicator ASVs. **f** Normalised ASV richness, with 5-point running average. **g** Differentiation index, which shows statistically significant changes in diversity as red dots. Background blue shading are meltwater pulses (MW1–4)^33^. The Younger Dryas (YD), the Bølling-Allerød (B-A) and Heinrich Stadial 1 (HS1) are indicated with dashed vertical lines. All plots show thousand years before 2000 AD (ka b2k).

Marine communities (Fig. 4d) during the early deglaciation (ca. 19–15 ka) were dominated by Cercozoans (46%), Fungi (17%) and Apicomplexa (9%). This changed around the B-A, when we recorded a community shift, which revealed four relative abundance peaks in the diatoms (from 50%, up to 90%) between ca. 14–8 ka.

Finally, an indicator analysis (see Methods) was used to identify open ocean and sea ice indicator ASVs and trace those in our record to reconstruct the environment. We used two calibration windows when the Labrador Sea was either a sea-ice free, open ocean (Holocene) or permanently sea ice covered (MIS 2) (Fig. 2f, g). The analyses identified five indicator ASVs in MIS 2 (20–35 ka) that were significantly associated with (near-)permanent sea ice cover. These sea ice ASVs were a Cryomonadida, two Stramenopiles, a parasitic alga and a green alga. We identified 194 ASVs to be significantly associated with open ocean environments of the Holocene (5–8 ka). The top five “open ocean” indicators were a polar centric diatom, two parasitic algae, a cercozoan, a Sagenista. When investigating the relative abundance of these five “sea ice” and the top five “open ocean” indicator ASVs (Fig. 2f, g) outside of the calibration windows, we observed strong indication for sea ice between 44 and 38 ka. While there is a clear drop in sea ice indicators during GI8 (37.5 ka), there was no increase in open water indicators. During the deglaciation, we observed an increase in sea ice ASVs, followed by an abrupt decline around 14 ka and four distinct pulses of open water ASVs (14–8 ka).

## Discussion

The changes in marine diversity and community shifts in the Labrador Sea around HE4 can be related to melting icebergs originating from a disintegrating Greenland and/or Laurentide Ice Sheet. Heterotrophic cercozoans, consumers in the microbial food web, comprised 70–80% of the community and thrived in the polar waters of GS9 (Fig. 3i). The Labrador Sea is considered to be covered by multi-year sea ice at this time^13^, but a small increase in primary productivity occurs around HE4, as reflected in the brassicasterol record (Fig. 3e) at the nearby site GS16-204-23CC (ca. 80 km to the east of our study site). Together with stable, low concentrations of sea ice biomarkers (Fig. 3f), this is more indicative for a dense sea ice cover with a short open-water season. A seasonal rather than a permanent sea ice cover, likely nearby the marginal sea ice zone, would indeed be a more plausible setting for melting icebergs and depositing IRD on the seafloor during HE4.

The major peak in IRD in the sediment of HE4 (Fig. 3d) indicates that icebergs melted in the Labrador Sea. Melting icebergs have been associated with increased nutrient supply and primary productivity^27,28^, which is reflected in the brassicasterol record^13^ but not in the foraminifer data^9^. The iceberg melting freshened the surface waters causing stronger stratification of the water column which can have a negative effect on planktonic foraminifer productivity^9^. Our data for HE4 showed a drop in richness as well as a significant shift in the community composition (Fig. 3g, h). The fresher surface waters likely promoted the increase in cercozoan Cryomonadida ASVs, a group which includes genera that indicate sea ice melt environments (e.g. *Cryothecomonas*^29^). Following the IRD peak, when the influence of fresh meltwater decreased again, we speculate that the observed increased marine diversity (Fig. 3d, g) was related to an increasing influence of nutrient-rich, warmer waters below the surface in an otherwise (seasonal) sea ice covered environment^9^.

At the onset of GI8, changes in marine diversity and community shifts in the Labrador Sea are caused by a change from a near-permanent to seasonal sea ice cover (Fig. 3c–f). The rapid climate warming at the beginning of GI8 caused a northward retreat of the polar front and our site became a highly productive seasonal sea ice environment influenced by arctic waters^9,13^ (Fig. 3c, e). While heterotrophic cercozoans were entirely dominant during GS9, their abundance declined considerably in GI8. Following the climate warming, diatoms (up to 24%) and Apicomplexa (up to 40%), became more abundant. The increase in Apicomplexa, which includes parasites of marine invertebrates, reflects a substantial change in the marine community and may also indicate weaker stratification^21^. It is likely that the site experienced unstable conditions for most of GI8, and a polynya environment or seasonal sea ice cover where warmer Atlantic-water is advected have both been proposed^9,13^.

The ocean–ecosystem changes are broadly contemporaneous with the rapid climate warming into GI8. However, they do not co-occur exactly and appear to happen at different rates. The NGRIP δ^18^O record^30^ shows a rapid warming over the Greenland ice sheet within decades (Fig. 3a), while the changes in the marine ecosystem appear to be more gradual and take place over centuries (Fig. 3g, h, j). The decline in species richness as well as the community shift started already in the latest GS9, i.e. prior to the abrupt climate warming. This indicates that ecosystem change was primarily controlled by ocean change, most likely due to subsurface warming^9^.

It is important to note that the sea ice indicator ASVs decrease in the early part of GI8, but only after the rapid warming event (Fig. 3j). The collapse of the sea ice indicator ASVs suggests that an important component of the sea ice community disappeared rapidly. We speculate that a shift occurred from a multi-year, dense and occasionally open sea ice cover to a seasonal sea ice setting, with more first year sea ice. Multi- and first-year sea ice environments are known to support different communities^5,31^, have higher productivity compared to full glacial conditions, and we speculate that our observations reflect such change. This aligns with the biomarker interpretation which indicates a seasonal, first-year sea ice environment^13^ and thus indicates that sea ice does not entirely disappear from the Labrador Sea during GI8 (Fig. 3f).

The marine communities change fundamentally during the deglaciation in the Labrador Sea (Fig. 4d–g), when the surface ocean transitions from a glacial, multi-year sea-ice-covered and low productive ocean^9^ to the warmer, nearly sea-ice-free, modern-like conditions of the Holocene. As the global climate and the surface ocean of the Labrador Sea warms during the last deglaciation^32,33^, the marine community became gradually more diverse between ca. 15 and 10 ka (Fig. 4f). The rapid warming at the onset of the B-A around 14.7 ka is reflected in the NGRIP δ^18^O record in Greenland and also in increasing sea surface temperatures in the Labrador Sea (Fig. 4a, b). However, this did not immediately leave an imprint on the Labrador Sea marine and sea ice communities – an observation also made for GI8.

Even after the rapid warming, the early B-A marine community (14.7–14 ka) remained dominated by cercozoans, fungi and apicomplexa. Such sea ice community is typical for most of the glacial and the LGM, when a permanent, multi-year sea ice cover occupied the Labrador Sea (Fig. 2e). Also the sea ice indicator ASVs remained in the assemblage until well after the rapid climate and Labrador Sea surface ocean warming (Fig. 4e). Our DNA data thus suggests that a substantial sea ice cover remained in the early B-A. Although not exactly aligning in timing, likely due to age model differences and sampling resolution, the IP_25_ sea ice biomarker remains also present in the earliest part of the B-A (Fig. 4c). At the same time, surface ocean temperature exceeds 4 °C during the B-A (Fig. 4b), indicating that summers are sea-ice-free but winters are not. We speculate that the sea ice reaching the site, most likely during winter, is multi-year sea ice that drifts into the Labrador Sea from the north and as such brings in the communities with cercozoans, fungi and apicomplexans.

Between 14 and 8 ka, we record major community shifts related to four subsequent meltwater pulses^33^ (MW1-4). All meltwater pulses have the potential to influence the surface ocean conditions, enhance primary productivity and influence marine communities^34^. Indeed, during each meltwater pulse a community shift is observed, and diatoms take up 50 to 90% of the community at our site (Fig. 4d, g). MW1 (14.1–12.9 ka) is characterised by oligotrophic, sea ice free, cold waters which remained above zero degrees Celsius (Fig. 4b, c) and these conditions were likely caused by fresh-water release from icebergs that are melting in the Labrador Sea^33^. The disappearance of the sea ice indicator ASVs and the increasing dominance of the open ocean indicator ASV (a polar-centric Mediophyceae diatom), supports the interpretation of the absence of sea ice during MW1. In contrast, meltwaters from MW2, MW3 and MW4 are suspension-rich and sourced from the disintegrating Laurentide and Greenland ice sheets^33^. These three meltwater pulses are characterised by increased sea ice formation, an abrupt decrease in sea surface temperatures and more oligotrophic conditions (Fig. 4b, c). The sea ice is likely formed locally and seasonally. This we interpret based on the absence of sea ice indicator ASVs (Fig. 4e), which are most likely indicating multi-year sea ice settings. Diatoms dominate the abundance peaks in each meltwater pulse, with a last peak occurring around 8 ka (Fig. 4d). Subsequently, between 8 and 5 ka, the Labrador Sea became entirely sea ice free, marginally warmer than the modern annual average (around 6–8 °C) and showed elevated productivity^33^. Here, alpha diversity was highest in our entire record, suggesting that warm open waters maintained a rich community.

Today, Arctic multi-year sea ice decreases rapidly and is being replaced by first-year sea ice, leaving a strong imprint on the Arctic marine communities^5^. While we acknowledge that the Labrador Sea is not the Arctic Ocean, the effect of the rapid change from permanent to seasonal sea ice conditions on the marine communities provides important insights for the current and future Arctic sea ice and the ecosystem it supports. What is common between the marine community changes around the rapid global warming events of GI8 and the B-A, is that these changes are only broadly contemporaneous to the timing of the rapid climate events. In both cases, the changes in marine communities appeared more gradual, on century rather than decadal scale. A community shift may have already been initiated prior to the rapid warming of GI8, due to changing conditions in the surface ocean, with first a freshening due to melting icebergs followed by (sub-)surface warming^9^. For both events, the community shifts were only fully expressed after the rapid warming but were unequivocally tied to the changing sea ice conditions.

The observations from the paleo-record thus provide a unique window on cryosphere–ecosystem links, and even on how this can impact global climate. Looking beyond the Labrador Sea during the B-A period, major community shifts are also reported in the Southern Ocean in relation to changing sea ice conditions: a shift to diatom-dominated communities occurred in the Scotia Sea^15^ and a dominance of the haptophyte *Phaeocystis antarctica* was reported close to the Antarctic Peninsula^35^. Such drastic changes to the dominant taxa in marine communities alter ecosystem functioning, pelagic-benthic coupling^21,36,37^ and the efficiency of the biological carbon pump in the (sub)polar regions, key regions for global climate^35,38^. Depending on which organisms become dominant (diatoms, *Phaeocystis antarctica*, parasites), this can increase or decrease the biological carbon pump and provide an important feedback from the ecosystem to the climate system. This feedback, the functioning of the biological pump, needs to be constrained better in paleo-records if we want to be able to project the impact of changing sea ice conditions and ecosystems in our future Arctic.

## Online methods

### Materials

During an expedition with RV G.O. Sars in 2016, two calypso cores were taken at Eirik Drift Station GS16-204-22CC (58° 02.83 N, 47° 02.36 W, water depth 3160 m). GS16-204-22CC-A (hereafter 22CC-A) and GS16-204-22CC-B (hereafter 22CC-B) recovered Holocene to MIS 6 sediments^9,39^. For this study, we investigated the MIS 3 to middle Holocene interval between 465 and 70 cm depth of 22CC-B. This sediment core was frozen immediately after splitting the core onboard and kept frozen until sampling in our labs.

### Age model

The age model for core 22CC-B was established as follows: we first correlated the colour reflectance parameters a* of 22CC-B with 22CC-A (Fig. S1, Suppl. Table S1), which were measured using a hand-held Konica Minolta CM 600 d spectrophotometer. This correlation is independently confirmed by the IRD data from both cores for Heinrich Event 4, where after correlation, the IRD concentrations from 22CC-A^9^ and 22CC-B (this study) overlapped (Fig. S2, Supplementary Table S2). Subsequently, we transferred the 22CC-A age model to 22CC-B. The 22CC-A age model^9^ is based primarily on the correlation of the planktonic foraminiferal δ^18^O and δ ^13^C records between 22CC-A and PS2644-5^40^, and further supported by AMS ^14^C dates, tephra-layers and geomagnetic data. To improve age control in the Holocene, we added four more AMS ^14^C dates in the upper part of the 22CC-B, an interval not covered by the 22CC-A age model. The four dates from 22CC-B were measured at Beta Analytics on the planktic foraminifer *N. pachyderma* and calibrated with the IntCal v.0.3.1 R package using Marine13 calibration curve^41^, no additional reservoir correction (∆R = 0 ± 0 years), and added 50 years to get to b2k ages, i.e. before 2000 AD.

### Sampling

The sampling for *sed*aDNA analyses was guided by the preliminary work of Griem et al.^9^. We sampled at a higher resolution around the Heinrich Events, initially based on correlation of magnetic susceptibility records of both 22CC-A to 22CC-B. Samples for DNA analyses were collected before initiating any other proxy work, this to ensure that the samples were as pristine as possible for DNA work. The 22CC-B core was split onboard during the cruise, covered with plastic film, put in sealed storage tubes and transferred immediately to the freezer. The entire sections were kept frozen (-20 °C) until onshore sampling. Sampling took place in a lab, where the bench space was cleaned thoroughly with bleach (10%) prior to sampling. The sections were gently thawed overnight (at 4 °C) before sampling. Samples were collected using sterile syringes (with cut-off tips) after the upper 2–5 mm of sediment was removed using sterile spatulas. Syringes filled with sediment were put into individual plastic bags and frozen again at −20 °C until DNA extraction.

All frozen sediments for *sed*aDNA analysis were handled in an access-restricted, positively pressurised room (HEPA filters) intended for nucleic acid extraction, while researchers wore appropriate personal protection equipment (single-use microfibre suits, particle masks, safety goggles and gloves). Room and equipment were thoroughly cleaned with a freshly-prepared 10% (v/v) solution of sodium hypochlorite in Milli-Q® water or DNAZap (Thermo Fisher Scientific). Sediment subsampling was done inside a class II laminar flow safety cabinet, whose internal surfaces were decontaminated using DNAZap (Invitrogen™ AM9890) and UV light. Prior to sampling, sediments were thawed at 4 °C for 1–2 h. The plunger was used to push the sediment out of the syringe, and we removed and discarded the 1 cm of exposed sediment using a sterile scalpel. Six individual subsamples of approximately 0.5 g (wet weight) were weighed directly into DNeasy PowerBead tubes (QIAGEN PowerSoil kit, Hilden, Germany) and frozen at −20 °C until DNA extraction with the DNeasy PowerSoil mini kit (QIAGEN) according to manufacturer instructions. All laboratory equipment and surfaces were cleaned again prior to processing the next sample.

### DNA extraction

DNA extraction was conducted in the same handling room and safety cabinet where subsamples were taken. Bead-assisted mechanical lysis was performed in a Tissue Lyzer II (Precellys, France) using the following programme: 6000 rpm for 40 s, 2 min pause, 6000 rpm for 40 s, 2 min pause and 6000 rpm for 40 s. After purification, DNA was eluted in 100 µl kit elution buffer, divided into two aliquots of 50 µl and stored at −20 °C. One aliquot was maintained as archive, while the other aliquot was utilised as the working DNA stock and always handled inside a clean class II laminar flow safety cabinet.

To identify potential modern DNA contaminants in *sed*aDNA preparations, we performed both sampling and extraction controls. For sampling controls, PowerBead tubes (Axygen, Corning Life Sciences, New York, USA) were opened and placed in a tube rack inside the safety cabinet and left open for random time intervals during sediment subsampling (times were noted). These sampling controls were processed the same as sediment-containing samples for DNA extraction and PCR amplification. For extraction controls, PowerBead tubes containing no sediment material were run through the DNA extraction protocol exactly the same as sediment samples. Eluates from DNA extraction for both sampling and extraction controls were stored at −20 ° C until PCR amplification.

We PCR-amplified the V7 hypervariable region of the small subunit ribosomal RNA (SSU rRNA) gene using universal eukaryote primers^42^. Sequencing libraries were prepared using a two-step PCR protocol. The first PCR targeted the V7 SSU rRNA gene region using primers F1183mod (5’ - AATTTGACTCAACRCGGG - 3’) and R1443mod (5’- GRGCATCACAGACCTG - 3’) that were appended with Illumina adapters and 12 N diversity stretches, as previously described^16^. Fifty microliter PCR reactions consisted of 1U of Phusion® High Fidelity Polymerase (Thermo Fisher Scientific), 1X HF buffer (Thermo Fisher Scientific), 250 nM final concentration of each primer, 0.2 mM final concentration of each dNTP, 0.2 mg/ml molecular biology grade Bovine Serum Albumin (New England Biolabs, USA), 5 µL template DNA, and ultrapure water. The amplification programme consisted of an initial denaturation at 95 °C for 5 min, followed by 30 cycles of 95 °C for 20 s, 59 °C for 30 s, 72 °C for 30 s, and a final elongation at 72 °C for 10 min. Amplification success and amplicon size (expected to be ranging from 260 to 360 bp) were determined by 1.5% (w/v) agarose gel electrophoresis with 1X GelRed® (Biotium, USA) staining. Amplicons were purified with magnetic beads (MagBio, USA) using a product:bead volumetric ratio of 1.8 in the first round, followed by a second round of purification using a volumetric ratio of 0.8. Purified amplicon concentration was measured with a Qubit fluorometer (Thermo Fisher Scientific, USA).

The second PCR ligated unique combinations of multiplex identifiers (8 unique forward primers (D501-D508) and 12 unique reverse primers (D701-D712)) and Illumina sequencing primer sequences onto the purified amplicons from the first PCR. Fifty-microliter PCR reactions consisted of 1U Phusion® HF Polymerase, 1X HF buffer, 0.2 mM final concentration of each dNTP, 5 µL template (diluted to 107 purified amplicons from the first PCR) and ultrapure water to 50 µL reaction volume. Each reaction had a unique combination of barcode forward (D5) and reverse (D7) primers, with concentration of 10 µM for forward and 5 µM for reverse. The amplification programme followed: initial denaturation at 95 °C for 5 min; 15 cycles of 95 °C for 20 s, 62 °C for 30 s, 72 °C for 30 s; a final elongation at 72 °C for 10 min. PCR products were visualised by agarose gel electrophoresis and purified with two rounds of magnetic beads using a product: bead ratio of 0.8 for both rounds. PCR products with visible residual primers, or primer dimers, were purified a third time. The concentration of purified amplicons was measured with Qubit and dual-indexed amplicons were pooled in equimolar ratios into a single sequencing library. The library was then purified twice using magnetic beads, first with a volumetric ratio of 0.8 then with a ratio of 1. Purified libraries were visualised using agarose gel electrophoresis and quantified by Qubit and NanoDrop One. The libraries were sequenced at the Norwegian Sequencing Center at the University of Oslo, Norway on an Illumina MiSeq platform using v.3 chemistry and 600 cycles (300 bp x 2). Demultiplexed sequence data was transferred to the Norwegian e-Infrastructure for Life Sciences and archived to the StoreBioInfo repository of the Norwegian Bioinformatics Platform (NorStore/NIRD).

### Bioinformatics

We first trimmed the primers sequences from the raw sequencing data using the cutadapt v3.4 software^43^ and discarded any reads that would not contain both the forward and reverse primers (no mismatch allowed). We then used the DADA2 v1.6 R package^44^ with default settings to filter poor quality reads (*filterAndTrim* function), the training of errors models (*learnErrors* function), the inference of Amplicon Sequence Variants (ASVs) with the *dada* function, the merging for paired-end reads (*mergePairs* function), the generation of the ASVs-to-sample matrix (*makeSequenceTable* function) and the filtering of chimeric ASVs (*removeBimera* function). We then taxonomically annotated the ASVs using the PR2 database^45^ and the assignment-fasta-vsearch module of the SLIM application^46^ that wraps the vsearch toolkit^47^. ASVs were annotated using a Last Common Ancestor approach, i.e. to the consensual taxonomic rank among up to three candidates reference sequences matching the query by at least 85% similarity. ASVs with 99% similarity or more with a reference sequence were directly annotated to that reference. We finally used the decontam R package^48^, with default settings, to identify and discard potential contaminants ASVs, by comparing their prevalence between in samples and negative controls (sampling and extraction controls).

We used the vegan v2.6-4 R package^49^ to calculate both alpha and beta diversity metrics. For alpha diversity, we calculated the Shannon index, where both richness (total species number) and evenness (relative abundance distribution) are accounted for (higher values means higher diversity), and the normalised richness (observed number of ASVs) by subsampling an even number of reads for each sample across the ASV matrix to account for difference in the number of sequences we obtained for each sample. We then normalised the ASV matrix using the Cumulative Sum Scaling method implemented in the metagenomeSeq v1.4 R package^50^, and computed a pairwise Bray-Curtis dissimilarity matrix. The Bray-Curtis distance matrix was used as input for a split moving-window analysis^25^ using the *smwda* function of the EcolUtils v0.1 R packages^51^ with default settings, to calculate a ‘differentiation index’ that can then be used to identify significant compositional shifts along the sediment record. Statistical significance was assessed using 1,000 permutations, with a threshold set at *p* < 0.05.

To identify ASVs that are associated with open ocean or sea ice environments, we used an indicator analysis. Based on the known Labrador Sea paleoceanography, the time interval between 3–8 ka is considered open ocean e.g.^26^, while between 20 and 35 ka the Labrador Sea environment was (near-)permanently sea ice covered e.g.^32^. We performed an indicator analysis using the *multipatt* function of the indicspecies v1.8.0 R package^52^ to identify ASVs as indicators (*p* < 0.05) for either open ocean or sea ice environments and concatenated their relative abundance to obtain a single vector of relative abundances of all open water or sea ice indicator ASVs (Figs. 3i, 4e). Because the indicator species are derived from the time intervals between 5–8 and 20–35 ka, those time intervals cannot be used within those time windows (see Fig. 2f, g). We only interpret the presence and abundance of these indicator species in the time window between 8–20 and >35 ka.

### Stable isotopes and ice rafted detritus (IRD)

We present new stable isotope data for the Holocene of 22CC-A (Fig. 2b) which extends the published MIS 3 record^9^. Samples for IRD analyses in 22CC-B around HE4 were sieved at different fractions, and grains were counted from the fraction >150 µm. Both the stable isotope analyses and IRD methodology are described in ref. ^9^.

### Reporting Summary

Further information on research design is available in the Nature Portfolio Reporting Summary linked to this article.

## Supplementary information

Transparent Peer Review file

Supplementary Information

NR Reporting Summary

## Data Availability

Sequence data and sample metadata is available on the European Nucleotide Archive repository via accession number PRJEB112075. Colour and stable isotope data from GS16-204-22CC-A; colour, ice rafted debris, radiocarbon, and paleogenomic data from GS16-204-22CC-B are available from PANGAEA^53^ at https://doi.org/10.1594/PANGAEA.994374. In this study we used previously published data which is available from https://doi.org/10.1594/PANGAEA.903936 (GS16-204-22CC-A), https://doi.org/10.1594/PANGAEA.106533 (HU90-013-013), https://doi.org/10.1594/PANGAEA.952329 (MSM12/2-5-1) and https://doi.org/10.5281/zenodo.7178400 (GS16-204-23CC) and https://doi.org/10.1594/PANGAEA.824867 (NGRIP).

## References

[1] Jahn, A., Holland, M. M. & Kay, J. E. Projections of an ice-free Arctic Ocean. *N. Rev. Earth Environ.* **5**, 164–176 (2024).

[2] Notz, D. & Stroeve, J. The trajectory towards a seasonally ice-free Arctic Ocean. *Curr. Clim. Change Rep.* **4**, 407–416 (2018). 30931246 10.1007/s40641-018-0113-2 PMC6411203

[3] Stroeve, J. & Notz, D. Changing state of Arctic sea ice across all seasons. *Environ. Res. Lett.* **13**, 103001 (2018).

[4] Brandt, S., Wassmann, P. & Piepenburg, D. Revisiting the footprints of climate change in Arctic marine food webs: an assessment of knowledge gained since 2010. *Front. Mar. Sci*. **10**, 1096222 (2023).

[5] Hop, H. et al. Changes in sea-ice protist diversity with declining sea ice in the Arctic Ocean from the 1980s to 2010s. *Front. Mar. Sci*. **7**, 243 (2020).

[6] Wassmann, P., Duarte, C. M., Agustí, S. & Sejr, M. K. Footprints of climate change in the Arctic marine ecosystem. *Glob. Change Biol.* **17**, 1235–1249 (2011).

[7] Flores, H. et al. Sea-ice decline could keep zooplankton deeper for longer. *Nat. Clim. Chang*. 1–9 10.1038/s41558-023-01779-1 (2023).

[8] Lannuzel, D. et al. The future of Arctic sea-ice biogeochemistry and ice-associated ecosystems. *Nat. Clim. Chang.* **10**, 983–992 (2020).

[9] Griem, L., Voelker, A. H. L., Berben, S. M. P., Dokken, T. M. & Jansen, E. Insolation and glacial meltwater influence on sea-ice and circulation variability in the Northeastern Labrador sea during the last glacial period. *Paleoceanog. Paleoclimatol.* **34**, 1689–1709 (2019).

[10] Pflaumann, U. et al. Glacial North Atlantic: sea-surface conditions reconstructed by GLAMAP 2000. *Paleoceanography* **18**, 2002PA000774 (2003).

[11] Sadatzki, H. et al. Sea ice variability in the southern Norwegian Sea during glacial Dansgaard-Oeschger climate cycles. *Sci. Adv.* **5**, eaau6174 (2019). 30854427 10.1126/sciadv.aau6174 PMC6402855

[12] Jansen, E. et al. Past perspectives on the present era of abrupt Arctic climate change. *Nat. Clim. Chang.* **10**, 714–721 (2020).

[13] Scoto, F. et al. Sea ice fluctuations in the Baffin Bay and the Labrador Sea during glacial abrupt climate changes. *Proc. Natl. Acad. Sci. USA* **119**, e2203468119 (2022). 36279448 10.1073/pnas.2203468119 PMC9636944

[14] de Vernal, A., Gersonde, R., Goosse, H., Seidenkrantz, M.-S. & Wolff, E. W. Sea ice in the paleoclimate system: the challenge of reconstructing sea ice from proxies – an introduction. *Quat. Sci. Rev.* **79**, 1–8 (2013).

[15] Armbrecht, L. et al. Ancient marine sediment DNA reveals diatom transition in Antarctica. *Nat. Commun.* **13**, 5787 (2022). 36184671 10.1038/s41467-022-33494-4 PMC9527250

[16] De Schepper, S. et al. The potential of sedimentary ancient DNA for reconstructing past sea ice evolution. *ISME J.* **13**, 2566–2577 (2019). 31235841 10.1038/s41396-019-0457-1 PMC6776040

[17] Lejzerowicz, F. et al. Ancient DNA complements microfossil record in deep-sea subsurface sediments. *Biol. Lett.* **9**, 20130283 (2013). 23658006 10.1098/rsbl.2013.0283 PMC3730641

[18] Zimmermann, H. H. et al. Marine ecosystem shifts with deglacial sea-ice loss inferred from ancient DNA shotgun sequencing. *Nat. Commun.* **14**, 1650 (2023). 36964154 10.1038/s41467-023-36845-x PMC10039020

[19] Seidenkrantz, M.-S. Benthic foraminifera as palaeo sea-ice indicators in the subarctic realm – examples from the Labrador Sea–Baffin Bay region. *Quat. Sci. Rev.* **79**, 135–144 (2013).

[20] Boetius, A. et al. Export of Algal Biomass from the Melting Arctic Sea Ice. *Science* **339**, 1430–1432 (2013). 23413190 10.1126/science.1231346

[21] Ramondenc, S. et al. Unveiling pelagic-benthic coupling associated with the biological carbon pump in the Fram Strait (Arctic Ocean). *Nat. Commun.* **16**, 840 (2025). 39833152 10.1038/s41467-024-55221-x PMC11747630

[22] Gloor, G. B., Macklaim, J. M., Pawlowsky-Glahn, V. & Egozcue, J. J. Microbiome datasets are compositional: and this is not optional. *Front. Microbiol*. **8**, 2224 (2017). 10.3389/fmicb.2017.02224 PMC5695134 29187837

[23] Greenacre, M., Martínez-Álvaro, M. & Blasco, A. Compositional data analysis of microbiome and any-omics datasets: a validation of the additive logratio transformation. *Front. Microbiol*. **12**, 727398 (2021). 10.3389/fmicb.2021.727398 PMC8561721 34737726

[24] Grant, D. M. et al. Sedimentary ancient DNA sequences reveal marine ecosystem shifts and indicator taxa for glacial-interglacial sea ice conditions. *Quat. Sci. Rev.* **339**, 108619 (2024).

[25] Ludwig, J. A. & Cornelius, J. M. Locating discontinuities along ecological gradients. *Ecol* **68**, 448–450 (1987).

[26] Solignac, S., de Vernal, A. & Hillaire-Marcel, C. Holocene sea-surface conditions in the North Atlantic—contrasted trends and regimes in the western and eastern sectors (Labrador Sea vs. Iceland Basin). *Quat. Sci. Rev.* **23**, 319–334 (2004).

[27] Romero, O. E., LeVay, L. J., McClymont, E. L., Müller, J. & Cowan, E. A. Orbital and suborbital-scale variations of productivity and sea surface conditions in the Gulf of Alaska During the Past 54,000 Years: impact of iron fertilization by icebergs and meltwater. *Paleoceanog. Paleoclimatol.* **37**, e2021PA004385 (2022).

[28] Barkley, A. E. et al. Patagonian dust, Agulhas Current, and Antarctic ice-rafted debris contributions to the South Atlantic Ocean over the past 150,000 years. *Proc. Natl. Acad. Sci. USA* **121**, e2402120121 (2024). 39042680 10.1073/pnas.2402120121 PMC11295081

[29] Thaler, M. & Lovejoy, C. Distribution and diversity of a protist predator *Cryothecomonas* (Cercozoa) in Arctic Marine Waters. *J. Eukaryot. Microbiol.* **59**, 291–299 (2012). 22703332 10.1111/j.1550-7408.2012.00631.x

[30] Bazin, L. et al. AICC2012 chronology for ice core NGRIP. PANGAEA 10.1594/PANGAEA.824867 (2013).

[31] Comeau, A. M. et al. Protists in Arctic drift and land-fast sea ice. *J. Phycol.* **49**, 229–240 (2013). 27008512 10.1111/jpy.12026

[32] Rahman, A. & de Vernal, A. Surface oceanographic changes in the eastern Labrador Sea: nannofossil record of the last 31,000 years. *Mar. Geol.* **121**, 247–263 (1994).

[33] You, D. et al. Last deglacial abrupt climate changes caused by meltwater pulses in the Labrador Sea. *Commun. Earth Environ.* **4**, 1–12 (2023).37325084

[34] Oliver, H. et al. Exploring the potential impact of Greenland meltwater on stratification, photosynthetically active radiation, and primary production in the Labrador Sea. *J. Geophys. Res. Oceans* **123**, 2570–2591 (2018).

[35] Weiß, J. F. et al. Carbon drawdown by algal blooms during Antarctic Cold Reversal from sedimentary ancient DNA. *Nat. Geosci.* **18**, 901–908 (2025).

[36] Braeckman, U. et al. Carbon and nitrogen turnover in the Arctic deep sea: in situ benthic community response to diatom and coccolithophorid phytodetritus. *Biogeosciences* **15**, 6537–6557 (2018).

[37] Lee, D.-G., Kwon, E. Y., Kam, J. & Kug, J.-S. Irreversible phytoplankton community shifts over Subpolar North Atlantic in response to CO_2_ forcing. *Earth Syst. Dyn.* **16**, 2101–2111 (2025).

[38] Cabrera-Brufau, M., Arin, L., Sala, M. M., Cermeño, P. & Marrasé, C. Diatom dominance enhances resistance of phytoplanktonic POM to mesopelagic microbial decomposition. *Front. Mar. Sci*. **8**, 683354 (2021).

[39] Steinsland, K. et al. Sea ice variability in the North Atlantic subpolar gyre throughout the Last Interglacial. *Quat. Sci. Rev.* **313**, 108198 (2023).

[40] Voelker, A. H. L. & Haflidason, H. Refining the Icelandic tephrachronology of the last glacial period – The deep-sea core PS2644 record from the southern Greenland Sea. *Glob. Planet. Change* **131**, 35–62 (2015).

[41] Reimer, P. J. et al. IntCal13 and Marine13 radiocarbon age calibration curves 0–50,000 years cal BP. *Radiocarbon* **55**, 1869–1887 (2013).

[42] Ray, J. et al. Molecular gut content analysis demonstrates that *Calanus* grazing on *Phaeocystis pouchetii* and *Skeletonema marinoi* is sensitive to bloom phase but not prey density. *Mar. Ecol. Prog. Ser.* **542**, 63–77 (2016).

[43] Martin, M. Cutadapt removes adapter sequences from high-throughput sequencing reads. *EMBnet. J.* **17**, 10–12 (2011).

[44] Callahan, B. J. et al. DADA2: high-resolution sample inference from Illumina amplicon data. *Nat. Methods* **13**, 581–583 (2016). 27214047 10.1038/nmeth.3869 PMC4927377

[45] Guillou, L. et al. The Protist Ribosomal Reference database (PR2): a catalog of unicellular eukaryote Small Sub-Unit rRNA sequences with curated taxonomy. *Nucleic Acids Res.* **41**, D597–D604 (2012). 23193267 10.1093/nar/gks1160 PMC3531120

[46] Dufresne, Y., Lejzerowicz, F., Perret-Gentil, L. A., Pawlowski, J. & Cordier, T. SLIM: a flexible web application for the reproducible processing of environmental DNA metabarcoding data. *BMC Bioinform.* **20**, 88 (2019). 10.1186/s12859-019-2663-2 PMC6381720 30782112

[47] Rognes, T., Flouri, T., Nichols, B., Quince, C. & Mahé, F. VSEARCH: a versatile open source tool for metagenomics. *PeerJ* **4**, e2584 (2016). 27781170 10.7717/peerj.2584 PMC5075697

[48] Davis, N. M., Proctor, D. M., Holmes, S. P., Relman, D. A. & Callahan, B. J. Simple statistical identification and removal of contaminant sequences in marker-gene and metagenomics data. *Microbiome* **6**, 226 (2018). 30558668 10.1186/s40168-018-0605-2 PMC6298009

[49] Oksanen, J. et al. Vegan: Community Ecology Package. https://CRAN.R-project.org/package=vegan (2022).

[50] Paulson, J. N., Stine, O. C., Bravo, H. C. & Pop, M. Differential abundance analysis for microbial marker-gene surveys. *Nat. Methods* **10**, 1200–1202 (2013). 24076764 10.1038/nmeth.2658 PMC4010126

[51] Salazar, G. EcolUtils: Utilities for community ecology analysis. https://github.com/GuillemSalazar/EcolUtils (2023).

[52] Cáceres, M. D. & Legendre, P. Associations between species and groups of sites: indices and statistical inference. *Ecol* **90**, 3566–3574 (2009). 10.1890/08-1823.1 20120823

[53] Felden, J. et al. PANGAEA – Data Publisher for Earth & Environmental Science. *Sci. Data* **10**, 347 (2023). 37268655 10.1038/s41597-023-02269-x PMC10238520

[54] Hillaire-Marcel, C. Stable isotope analysis on sediment core HU90-013-013. *PANGAEA* 10.1594/PANGAEA.106533 (2003).

[55] Rasmussen, S. O. et al. A stratigraphic framework for abrupt climatic changes during the Last Glacial period based on three synchronized Greenland ice-core records: refining and extending the INTIMATE event stratigraphy. *Quat. Sci. Rev.* **106**, 14–28 (2014).

