# Supplementary material for: Changing sea ice cover led to marine biodiversity shifts in the Late Quaternary
Source: Commun Earth Environ. 2026 Jun 4;7(1):732. doi: 10.1038/s43247-026-03696-5 (PMC13549946; doi:10.1038/s43247-026-03696-5)
Supplement: Supplementary file 1 [file 43247_2026_3696_MOESM1_ESM.pdf]

nature portfolio

Peer Review File

Changing sea ice cover led to marine biodiversity shifts in the Late Quaternary

Corresponding Author: Dr Stijn De Schepper

This manuscript has been previously reviewed at another journal. This document only contains information relating to versions considered at Communications Earth & Environment.

This file contains all editorial decision letters in order by version, followed by all author rebuttals in order by version.

Attachments originally included by the reviewers as part of their assessment can be found at the end of this file.

Version 0:

Decision Letter:

Dear Dr De Schepper,

Your manuscript titled "Marine community shifts at past rapid climate transitions" has now been seen by 2 reviewers, whose comments are appended below. In the light of their advice we regret to inform you that we cannot publish your manuscript in Communications Earth & Environment.

You will see that the reviewers raise substantive concerns. Importantly, they raise numerous concerns that we consider would be too substantial to address in a simple review regarding methods, appropriate use of references, and other issues around the interpretation of the data. Taking these points together with our editorial considerations, we are unable to conclude that your manuscript represents a sufficiently novel and compelling advance over the body of related work in the literature. Unfortunately, these reservations are sufficiently important to preclude publication of this study in Communications Earth & Environment.

We are sorry that we cannot be more positive on this occasion and thank you for the opportunity to consider your work.

Best regards,

Christopher Cornwall, PhD
Editorial Board Member
Communications Earth & Environment
orcid.org/0000-0002-6154-4082

Carolina Ortiz Guerrero, PhD
Associate Editor
Communications Earth & Environment

Reviewers' comments:

Reviewer #1 (Remarks to the Author):

Dear authors,

The submitted works from De Schepper et al., entitled “Marine community shifts at past rapid climate transitions” consists of original research on an important, yet undocumented effect of Arctic Sea ice cover loss on non-fossilized past biodiversity, using sedimentary ancient DNA (sedaDNA) for over 100 samples taken along a sediment core and extensive glaciology proxies to contextualize changes for micro-eukaryotic taxa that include major planktonic components. These innovative and robust features all make this work a novel contribution worthy of publication for a broad readership including

oceanographers, paleontologists, ancient molecular biologists, and potentially modelers interested in harnessing omics data to learn from past condition reconstructions.

As per my particular expertise on ancient sedimentary DNA, I believe that the high-quality of this contribution will influence thinking in this field, with its most-expected application to glacial-deglacial events reconstruction that's critical in the Arctic. Since my expertise lies not in the fields of glaciology or paleontology, my review and comments mainly deal with the sedaDNA molecular methods, bioinformatics and biostatistical analyses of the sequencing data.

To review its structure and assess redundancies, I broke down the manuscript to the paragraph level, each with a summary of the main conveyed message and main comments that I hope will be helpful. In view of this structure breakdown (see below), it appears that there is redundancy – especially in the Discussion section – which could be greatly reduced given the efficient and clear writing skills of the authors. Shortening the manuscript by aggregating the salient sedaDNA findings associated with glaciation-deglaciation events more directly and concisely, will but make this flawless work an even more impactful contribution to the field. Indeed, data amount and reported changes seem highly promising so that the authors could offer a revised version with moderate effort.

One effort yet concerns the statistical analysis, which consist in (i) describing alpha diversity increases/decreases, (ii) identifying community shifts, and beta diversity differentiation events using a suitable moving-window approach, and (iii) providing relative abundances for major taxa between (groups of) samples marking glaciation-deglaciation events. For (ii), more precision would be needed to support claims of "significant community shifts" (a result placed in the Discussion section, but not substantiated in the Results section): what test statistic was measured to claim significance? Also, the methods specify that "matrix was used as input for a Non-Metric Multidimensional Scaling (NMDS) using the metaMDS function", but this analysis is not presented: maybe it wouls help measuring significance levels? For (iii), it is important to consider that sequencing omics data is compositional (doi.org/10.3389/fmicb.2021.727398). This is especially true in this study where the Cercozoa component might include benthic ASVs whereas the diatoms ASVs are likely pelagic. Thus an observed decrease in relative abundance of diatoms might only be due to benthic cercozoans increasing in true abundance in the benthic sample, whereas the true abundance of diatoms would possibly not have changed, or even, increased in surface waters, but possibly not to a point that its sedaDNA would be more abundant than authochotonous benthic cercozoan DNA. This major drawback can be addressed by "considering pairwise ratios of the components and by analyzing these ratios after logarithmic transformation". This is quoted from the above DOI article, which proposed using ALR transform "the essential step being the choice of the reference component". Hence the authours could tackle both my comments by selecting a prevalent enough yet least variable (set of) ASV(s) to use a reference and that would enhance interpretability and highlight this key issue to the field of sedaDNA analysis. For example, strictly pelagic taxa/ASVs could serve as internal references for such log-ratios calculation and assert the reported changes at glaciation/deglaciation events. Such log-ratios could be computed only for the major events/shifts to provide support to the claims.

Introduction

§1 = Sea ice change events in the Arctic and in the Labrador Sea from 50 kyrs to now.
Main comment: "Several degrees on matter of decades" is somewhat approximative: could a more accurate number be given?

§2 = Impact on biodiversity and strength of sedaDNA to include the non-fossilized majority into the analysis.
Main comments:
- It would be good to provide more examples/names of taxa uncovered using sedaDNA. Indeed, "restricted view", "more complete picture", and "organisms that do not produce a shell, test or cyst" are too broad for the readership to grasp a sense of the large diversity that can be encompassed in sedaDNA analyses. Could be extracted from the cited papers, notably from that focused on the Arctic.
- It is important for those taxa belonging to phyla well known in the fossil record (e.g., soft-shelled forams) and it would be good if a sense of the large amount of non-fossilized diversity could be given.

§3 = Current influence and sea ice change in the Labrador Sea, incl. major events
Could "Labrador Sea" be labeled on Fig 1?
Main comments:
- There seem to be redundancy with the §1 where is clearly explained that: "the glacial Labrador Sea was characterised by stable, multiyear sea ice conditions during Greenland stadials (GS) whereas during the Greenland interstadials (GI) unstable, seasonally sea ice free conditions occurred", vs here in §3, that "During the Last Glacial, the Labrador Sea was mostly permanently sea ice covered, but during warm interstadials a seasonal sea ice cover occurred, and even open ocean conditions may have occurred during summer". To me this is the same, thus I recommend that the Intro section is slightly revised to introduce once only all the Labrador Sea changes relevant to the study. Also, are the three cited references in §3 all pointing to the same fact, or could there be some additional relevant details to more accurately extract from this literature? This §3 should provide a brief account of the question addressed more precisely, of the data constituted for this research, of the expected results and extended scope for a broad readership.
- "During the deglaciation […] during the Holocene": as constructed, these durations are not clear, please could approx. times be indicated or the sentence cut into two? Also, this continued account of key events would best fit before explaining the sedaDNA approach as it seems, is a logical continuation of §1. Again, the §3 could be more focused on what is unknown and that could help advance knowledge about past sea ice cover changes, how including more biodiversity can help, and what is done here (and expected) to make such advance.

Results

The whole section consists of a single paragraph: the first half relates the methods and sedaDNA sequencing yields. The other half superficially presents the taxa, their dominance patterns derived from relative abundances and that diversity shifts were identified that coincide with sea ice cover transition events. It represents a "teaser" for the actual results that are well elaborated in the following Discussion section. Indeed, the brevity of the Results section is surprising as the Discussion section is lengthy and substantiated with many results, (e.g., "During GI8, alpha diversity…"). Hence, I encourage the authors to merge everything into a Result & Discussion section (if agreed with editors), or to elaborate with results sorted off the Discussion.

Either way, slightly more taxonomic details would be useful to identify the ASVs of likely pelagic origins to use in the analyses, as only these ASVs would provide the non-"restricted view on communities and biodiversity of the water column". Re-running the analyses based on a (likely pelagic) subset of the ASVs would represent relatively little work, and provide the sedaDNA community with an highly-relevant, rare comparison.

Discussion

§1 = alpha diversity high (vs low) in deglacial (vs glacial) conditions
Main comments:
- "clearest increase": which statistic based on alpha diversity measures supports this claim?
- the message that "alpha diversity is associated with highly variable ocean conditions, where both sea ice and warm Atlantic waters can occur simultaneously or sequentially" is not a very clear. Does it mean that alpha diversity and ocean conditions are correlated?

§2 = beta diversity, community changes occur at major events where the abundance of major taxa increase/decrease; sampling rosolution is an issue but not at HE4.
Main comments:
- "significant community shifts": which statistic based on beta diversity measures supports this claim?
- statistically robust results about the changes in reported taxa abundances are missing in the Results section.

§3 = alpha, beta and taxa changes along highly resolved GI-GS and water freshening shifts.
- Redundancy of the topic but seemingly contradicting with respect to alpha diversity, between "In the late part of GS9, melting icebergs release IRD to the sediment (HE4), and the surface waters become fresher (Fig. 3d). At peak IRD release, we record a decrease in alpha diversity", and:
(1). "Alpha diversity bounces back rapidly to increase towards the end of the GS9" (§3)
(2). "Consistent high alpha diversity is recorded at the end of GS9, following the melting of icebergs in the Labrador Sea as shown in the IRD record (Figure 3d)" (§1)
- "shift is mostly driven by": any formal statistical measure?
- Also, it seems essential that the "GI", "GS", "HE" periods/events are directly indicated on all plots as for figure 1 ("Greenland Stadials (GS) in black, Greenland Interstadials (GI) in red)": these are missing form figure 3, making it hard to read alongside the text.
- Also, please homogenize "Fig." vs "Figure" notation style.

§4 = alpha, beta and taxa increase at GI8-GS9 transition as sea ice recedes.
Redundancies between "at the transition from GS9 to GI8, we record a decrease in alpha diversity and a community shift when the multi-year sea ice cover is rapidly replaced by seasonal sea ice" and:
(1). "In the late part of GS9, melting icebergs release IRD […] At peak IRD release, we record a decrease in alpha diversity and a significant shift" (§3)
(2). "Consistent high alpha diversity is recorded at the end of GS9, following the melting of icebergs in the Labrador Sea as shown in the IRD record and the rapid warming at the GS9-GI8 transition" (§1)
This is another example of what makes the discussion too verbose, because it scatters the same messages in multiple paragraphs. Note that I am not reporting any further instance of redundancy, as this is the authors' task to carefully revise.

§5 = low alpha diversity and (unspecified) beta changes, and taxa increase at GI8-GS8 transition
Main comment:
- "diatoms and Apicomplexa became more abundant at the expense of Cercozoa": this would necessitate log-ratio comparions between the groups of samples before and after this "becoming".

§3, §4 and §5 seem to describe in detail a series of events that rapidly succeed one another between 39 and 3 ba b2k (since the resolution is higher for this period), but the level of redundancy amongst them and with §1 is too high.

§6 = More recent warm-water ice melting drives increase in alpha and diatoms abundance; alpha high as sea ice recedes.
Main comments:
- "This changes during the B-A, when the first of a series of meltwater pulses is recorded": the meaning of "This changes" is not clear. Is it about the dominance patterns? For all taxa? And to what result does it changes?
- High verbosity of "increasing Greenland and Labrador Sea temperatures and decreasing sea ice cover" followed by similar meaning of "seasonal and marginal sea ice […] gradually becomes more influenced by warmer Atlantic waters". I would suggest shortening to the essential: discussing results.
- "increase in diatom ASV abundance": please use compositional data analysis to confirm this or heavily discussion th

aforementioned shortcoming about relative abundance issues and the fact that sedaDNA could in fact just have less benthic DNA on the rest of the relative abundance total.

§7 = Even more recent warm-water ice melting drives increase in alpha and diatoms abundance; possuble change of the benthic community;
Main comments:
- “alpha diversity gradually increases (Fig. 4g) with a disappearing sea ice cover and increasing Labrador Sea temperatures as the region becomes more influenced by warmer Atlantic waters”. While this is from another time period (yet not clearly bounded as “during deglaciation”), this is yet another similar verbose sentence documeting the same patterns above, i.e., some level of redundancy.
- Also redundant: “The late glacial and early deglacial up to the Bølling-Allerød marine communities were dominated by Cercozoans, Radiolaria and Apicomplexa” and from §6 “Prior to the Bølling-Allerød (B-A), the marine community was dominated by Cercozoans, Radiolaria and Apicomplexa”
- Other sentences are exactly redundant with the rprevious paragraph. My opinion is that such systematic repeats but for different periods would best be presented using a table.
- “Here, alpha diversity is highest in our entire record, indicating that warm open waters maintain a rich community” repeats §6 “Here the alpha diversity is highest in our entire record, indicating that warm, sea ice free waters maintain a rich community”.
In fact, it seems that the authors made a mistake when submitting, a version with duplicated subsections…

§8 = reflection on todays’ changes and the strenght of sedaDNA
Main comment: owing to my main comment about the superficiality of the results at high-level taxa and the lack of scrutiny to distinguish benthjic vs “surface ocean biosphere” taxa makes the following claim an overstatement: “We documented the effect of climate change and a changing sea ice environment on the surface ocean biosphere, and our metabarcoding study firstly demonstrates that DNA stored in Late Quaternary marine sediments can document past community changes.” Moreover, the fact that non-fossilized taxa that 18S primer will recover is not a novel demonstration.

§9 = highlights of main events translated into diversity changes and their frequency
No comment as this is the key relevant and strength of this work and should be the focus of more elabiorate result and impactful discussion, that could also cite previous sedaDNA results for other events/changes.

§10 = the global impact of diatom surges is ice free waters
Main comment: I feel that this last paragraph is too verbose to convey the intuitive activation of biological pumps in ice free conditions, and thus loses the focus on community-wide shifts (i.e., the title). I would even suggest reducing text about this mechanisms to two sentences dealing with the Southern Ocean example on the importance of a taxon like diatoms to model deglaciation impacts on atmCO2 and moving them in the Introduction section. Thus, the Discussion section could more briefly reflect the diatom increase results onto this mechanism, ot then speculate and invite for further high-resultion sedaDNA research on climate feedback at glacial-degracial interfaces.
- “a shift to a diatom-dominated ecosystem shift” is not clear.

Quite clearly, the main messages is of higher/increased alpha diversity and beta diversity shifts when sea ice recedes. Because it is scattered across multiple paragraphs, this trend is more difficult to grasp. Hence, my suggestion of increasing the results section with tests to better assert these changes, and of reducing the discussion to explain this trend once, using all the different sea ice change events as systematic, concurring evidence.

Minor comments

I note that line numbers are missing which complicated the reporting of minor comments. I expect the authors to be able to find out where the cited are located:
- cut into two sentences: "During Greenland stadials (GS) that occurred between ca. 50,000 and 25,000 years ago, the glacial Labrador Sea was characterised by stable, multiyear sea ice conditions. However, during Greenland interstadials (GI) it was unstable and seasonally free of sea ice."; or it changes meaning?
- "On Greenland" refer to the terrestrial biome, correct? Please specify as the sentence also
- "during stadial to interstadial transitions": would it work to use the acronyms? "during GS-GI transitions"?
- “was replaced by seasonal sea ice to open water conditions”: please specify if meaning “by seasonal sea ice and then by open water conditions”? Or “by seasonal sea ice or open water”? (I note a frequent use if “conditions”, is it necessary?)
- To cut verbosity, would this retain meaning as on-going and future?: “These events are relevant the understand sea ice change in the Arctic. Here a transition from ice-covered to ice-free ocean is ongoing and its measured effect of __<reducing?
>__ Arctic Ocean biodiversity will likely continue in the future”:
* There, are the “white” and “blue” labels necessary since this is not extensively referred to thereafter (blue is used in the figures, but legends suffice).
* Changed “sea ice free ocean” as it is somewhat redundant
* Also, could the nature/general trend of the reported “imprint on Arctic Ocean biodiversity already today” be clarified (see suggestion)?

This research does not raise any particular ethical concern but the code could be made available if possible. The authors de provide all details so that experiments could be reproduced based whence the data is made available. I have no doubt that the authors will indeed provide the raw sequencing data and metadata in public repositories.

Good luck!

Reviewer #2 (Remarks to the Author):

De Schepper and co-authors are presenting sedaDNA results for glacial to Holocene sections of a sediment core recovered off the southern tip of Greenland. Those are the first results for that region focusing on the eukaryotes in general during those particular periods. The authors observe clear changes in the plankton (?) ecosystem composition along with the different background climate scenarios and indicate that their results could provide insights into the future evolution of the Arctic Ocean. The results merit publication, but the current manuscript is in no shape to be published and, in my opinion, does the data not justice. I was very exited reading the manuscript title and the abstract, but then ended up underwhelmed when reading the discussion, which is very descriptive and short on “ecosystem scenarios”. The manuscript is doing a poor job bridging between the different disciplines leaving the reader a bit lost. For example, with my background in paleoceanography some of the eukaryotes groups mentioned are unknowns and the authors fail to mention if all those groups are plankton, what size plankton, an important information to know where in the ecosystem functioning food chain they are relevant, or if they are common in the modern subpolar/polar ocean. For someone working in marine biology/ecosystem function, the description “the ecoystem changes” would probably not be enough as I would like to know in what direction/to which ecoregion etc. (and thus potentially what such a change could mean for the ecosystem in general, including fishes and mammals). In this context, I am missing any comparison with Tara Ocean genomics results (or others) that could help to describe if the encountered eukaryote groups are typical for the Labrador Sea, the Nordic Seas or the Arctic Ocean, so that observed past ecosystem (well, in reality only a small part of the ecosystem) changes could be related to present day oceanographic conditions. I was also hoping for a better integration with existing palaeoecology data from the region, such as the planktonic foraminifer abundance data from the Griem et al. study at the same location or microfossil evidence from the wider region, especially regarding the diatom abundance peaks during the last glacial/interglacial transition. I put a related comment and potential publications to look at in a comment in the uploaded annotated manuscript. For example have indicators for higher productivity of diatoms counterparts in increased production in zooplankton? The recently published sedaDNA study (Grant et al., 2024; by the same group) for the previous interglacial period and preceding glacial in the same sediment core is doing a much better job in that perspective; a study, which is not even mentioned in the current manuscript.

Besides those major concern, I added comments in the annotated manuscript file, which I will not repeat here -with one exception: please use the correct species name of Neogloboquadrina pachyderma; see
https://www.mikrotax.org/pforams/cenozoic/Neogloboquadrina_pachyderma
https://www.marinespecies.org/aphia.php?p=taxdetails&id=113458

Unfortunately, I believe the manuscript should be rejected in its current, but the authors given the chance to come back with a substantially revised manuscript.

Here are some modern ocean references, which could help to improve the manuscript (although I did not check in detail):

Pierella Karlusich, J. J., F. M. Ibarbalz, and C. Bowler (2020), Phytoplankton in the Tara Ocean, Annual Review of Marine Science, 12(1), 233-265, doi: 10.1146/annurev-marine-010419-010706.
Ibarbalz, F. M., et al. (2019), Global Trends in Marine Plankton Diversity across Kingdoms of Life, Cell, 179(5), 1084-1097.e1021, doi: https://doi.org/10.1016/j.cell.2019.10.008.
Righetti, D., M. Vogt, N. Gruber, A. Psomas, and N. E. Zimmermann (2019), Global pattern of phytoplankton diversity driven by temperature and environmental variability, Science Advances, 5(5), eaau6253, doi: 10.1126/sciadv.aau6253.
Richter, D. J., et al. (2022), Genomic evidence for global ocean plankton biogeography shaped by large-scale current systems, eLife, 11, doi: 10.7554/eLife.78129.
Arrigo, K. R. (2014), Sea Ice Ecosystems, Annual Review of Marine Science, 6(1), 439-467, doi: 10.1146/annurev-marine-010213-135103.
Fragoso, G. M., A. J. Poulton, I. M. Yashayaev, E. J. H. Head, M. C. Stinchcombe, and D. A. Purdie (2016), Biogeographical patterns and environmental controls of phytoplankton communities from contrasting hydrographical zones of the Labrador Sea, Progress in Oceanography, 141, 212-226.
Fragoso, G. M., A. J. Poulton, I. M. Yashayaev, E. J. H. Head, G. Johnsen, and D. A. Purdie (2018), Diatom Biogeography From the Labrador Sea Revealed Through a Trait-Based Approach, Frontiers in Marine Science, 5, doi: 10.3389/fmars.2018.00297.
Mayot, N., P. A. Matrai, A. Arjona, S. Bélanger, C. Marchese, T. Jaegler, M. Ardyna, and M. Steele (2020), Springtime Export of Arctic Sea Ice Influences Phytoplankton Production in the Greenland Sea, Journal of Geophysical Research: Oceans, 125(3), e2019JC015799.

Version 1:

Decision Letter:

** Please ensure you delete the link to your author home page in this e-mail if you wish to forward it to your coauthors **

Dear Dr De Schepper,

Please allow us to apologise for the delay in sending a decision on your manuscript titled "Changing sea ice cover led to marine biodiversity shifts in the past". It has now been seen again by our reviewers, whose comments appear below. In light of their advice we are delighted to say that we are happy, in principle, to publish a suitably revised version in Communications Earth & Environment.

We therefore invite you to revise your paper one last time to address the remaining concerns of our reviewers. In particular, please ensure that you expand the reference list so it is more holistic, ensure consistency with nomenclature and acronyms, and fully discuss the role different taxonomic groups play in the ecosystem. At the same time we ask that you edit your manuscript to comply with our format requirements and to maximise the accessibility and therefore the impact of your work.

EDITORIAL REQUESTS:

Please review our specific editorial comments and requests regarding your manuscript in the attached "Editorial Requests Table".

*****Please take care to match our formatting and policy requirements. We will check revised manuscript and return manuscripts that do not comply. Such requests will lead to delays. *****

Please outline your response to each request in the right hand column. Please upload the completed table with your manuscript files as a Related Manuscript file.

If you have any questions or concerns about any of our requests, please do not hesitate to contact me.

SUBMISSION INFORMATION:

In order to accept your paper, we require the files listed at the end of the Editorial Requests Table; the list of required files is also available at https://www.nature.com/documents/commsj-file-checklist.pdf .

OPEN ACCESS:

Communications Earth & Environment is a fully open access journal. Articles are made freely accessible on publication. For further information about article processing charges, open access funding, and advice and support from Nature Portfolio, please visit https://www.nature.com/commsenv/open-access

At acceptance, you will be provided with instructions for completing the open access licence agreement on behalf of all authors. This grants us the necessary permissions to publish your paper. Additionally, you will be asked to declare that all required third party permissions have been obtained, and to provide billing information in order to pay the article-processing charge (APC).

Please use the following link to submit the above items:
Link Redacted
** This url links to your confidential home page and associated information about manuscripts you may have submitted or be reviewing for us. If you wish to forward this email to co-authors, please delete the link to your homepage first **

We hope to hear from you within two weeks; please let us know if you need more time.

Best regards,

Christopher Cornwall, PhD
Editorial Board Member
Communications Earth & Environment
orcid.org/0000-0002-6154-4082

Joe Aslin
Deputy Editor,
Communications Earth & Environment
Consulting Editor,
Communications Sustainability

https://www.nature.com/commsenv/
Twitter: @CommsEarth

REVIEWERS' COMMENTS:

Reviewer #2 (Remarks to the Author):

It is now the second time that I read the manuscript by de Schepper et al. reporting on sedaDNA results from the Labrador Sea. The authors made a great effort in revising the manuscript that reads much more fluent now, better integrates sedaDNA and paleoclimatic evidence and outlines implications. So, I congratulate the authors on the very effective revision and recommend publication of the manuscript subsequent to minor revision and some clarifications.

I make my comments below related to line numbers in the tracked changes word document version of the manuscript as I read that text. I am listing the three more critical comments first, with the remaining comments following then by order in the manuscript.

Line 293: you might want to be consistent in age related nomenclature throughout the manuscript, i.e., you use years in the introduction (and then again in lines 357 and 360) and then jump to ka here in the results. This should be really confusing for readers not familiar with the switch between years and ka, e.g., coming from a biological background.

Line 530: what is the reference for the meltwater pulses (note that you use MWP in the manuscript but only MW in figure 4)? If you identified them, please state based on what evidence because I am puzzled by the missing IP25 peak during MW 1.

Lines 599 and 1045, 1057, 1059 and figure 2 and 3: delete sinistral or (sin); the species in now just called N. pachyderma (as the formerly right coiling "variant" is now N. incompta); that also needs to be corrected in the axis labels of figures 2b, 3b and 3c = Np instead of Nps.

Introduction: include a sentence explaining why the Labrador Sea record can be used as a case study for future Arctic Ocean change, keeping in mind that the study sites is within the subpolar gyre and not experiencing truly arctic conditions (at least nowadays). It (finally) comes up in lines 193-195 and I suggest to move this sentence up into line 187 to make the case for the causal connection before you go into the paleoclimatic changes in the Labrador Sea.

360: check placement of callout to figure 2c
367: IRD has not been defined, yet. Given the multidisciplinary readership of Nature Communications, you might want to limit the number of acronyms you define; so, if you use IRD only a few times, I would recommend to spell it out.
368: I believe it should say "increases by …."
370: no, the Labrador Sea is not ice-free as both the East/West Greenland Currents and the Labrador Current carry icebergs and seasonally sea-ice. You need to be more precise here in your wording; maybe use something like central Labrador Sea or open ocean environment of the Labrador Sea.
386: check wording; I believe you meant to say something like "a site with higher sedimentation rates and denser (temporal) resolution sampling would be needed".
413 (line 150 in clean version pdf file): delete the bracket at the end of the sentence.
495: delete in after which
532: I believe it should say during each MWP instead of in…
541: was SST defined as acronym?

571 (or in 573): you could potentially cite also Kelly, S., E. Popova, A. Yool, F. Jebri, S. Oliver, and M. Srokosz (2025), Abrupt Changes in the Timing and Magnitude of the North Atlantic Bloom Over the 21st Century, Journal of Geophysical Research: Oceans, 130(3), e2024JC022284, doi: https://doi.org/10.1029/2024JC022284.
and/or Lee, D. G., E. Y. Kwon, J. Kam, and J. S. Kug (2025), Irreversible phytoplankton community shifts over Subpolar North Atlantic in response to CO2 forcing, Earth Syst. Dynam., 16(6), 2101–2111, doi: 10.5194/esd-16-2101-2025.

607: to me the phrasing here reads wrong. I would substitute "prior to" with "before" or if you want to keep prior to change "work was" to "work being". Also, delete this after the comma.

Figure 1: the blue arrow should be corrected within the Labrador Sea to reflect the pathway of the West Greenland Current that flows along the Greenland margin up to the Baffin Bay.

Figure 2: last two grey bars on the right hide the isotope records in panel b.

Reviewer #3 (Remarks to the Author):

De Schepper et al. provided a manuscript titled "Changing sea ice cover led to marine biodiversity shifts in the past". This work uses sedimentary ancient DNA (sedaDNA) in a calypso core retrieved in the Labrador Sea to reconstruct past changes in the marine eukaryotic communities, and to correlate these changes to marine cryosphere oscillations. Their findings suggest a strong relationship between changes in sea ice cover and distribution and ecosystem shifts, a link which was already hypothesized but only marginally studied in a paleorecord. Besides the important implications for ecology and biology of (sub)polar regions, this work brings also important insights for the "paleoreconstruction" community, showing how an innovative methodology can be used, coupled with more traditional proxies, to further constrain the past evolution of

marine environments.
The Authors went through a first revision and sensibly improved the manuscript. I have only a few comments that needs to be addressed and some minor revisions to make the text more reader friendly. Since during the first revision the sedaDNA methodology and statistical analysis was already thoroughly scrutinized, and since my expertise lies mainly in geochemistry and paleoreconstructions, I focused on the “paleo”-implications of the study and on the side proxies that were coupled to sedaDNA results.
GENERAL COMMENTS
1) Following a comment from previous Reviewer #2, the Authors added some details about the taxonomic groups they used, but I still find it difficult to visualize the different roles of these groups in the ecosystem. I suggest adding a table, maybe in the SI but referenced in the main text, with a brief description of the ecosystem niche/role of the main groups used in the discussion (Cercozoa, Apicomplexa, etc) and their main characteristics. I understand that these groups are not entirely homogenous (and also that, with this methodology, it is not easy to refer to genus or species level), but this is something worth specifying as well, for the sake of the reader.
2) The Authors make use of diversity indices, which are all already established in the literature, but it is nonetheless missing a description, in the Method section, of what these indices are and what they show.
3) I would suggest using the PIP25 index, combining brassicasterol and IP25 (Belt et al., 2018), to better visualize the intertwining of open water conditions and seasonal sea ice influence. The Authors use IP25 and brassicasterol to make more robust interpretation of sea ice vs open water influence, but it is challenging sometimes to correlate the abundance of single biomarkers to a certain environmental variable. For example, an increase in IP25 content does not directly imply an increase in seasonal sea ice influence, this is just one possible explanation, but it could also imply an overall increase in marine and sympagic productivity. The Authors made perfectly sound inferences, but nonetheless using the PIP index would massively help visualizing the changes in sea ice influence.
4) In Figure 2b the black triangles indicating AMS ages are missing. In Figure 3 an IRD record from core 22CC-B is shown as novel, but in the Method section there is no description of how this record was evaluated. In Figure 4d the color legend is missing.

SPECIFIC COMMENTS
Lines 20-24: I would slightly rephrase here: “Changes in diversity and communities happen slower than the rapid climate warming during Greenland Interstadial 8 (ca. 38,000 years ago) and the Bølling period (ca. 14,700 years ago). Surface ocean conditions, rather than atmospheric warming, seem to exert control on the ecosystem, and diversity changes are linked to (sub-)surface warming, a retreating or changing sea ice cover and freshening of the surface via meltwater pulses”
Line 35: change “impact of a change” with “impact of this change”
Lines 44-46: the replacement of a permanent sea ice cover with seasonal sea ice/open waters is referred to stadial/interstadial transitions?
Line 91: how are alpha and beta diversity patterns defined? Why do they have a different name as the one in the figure/caption? Fig. 2c refers to ASV richness, how are they related? See also general comment #2.
Line 103-104: higher diversity suggests a lower influence of differential preservation, is it just because more differential preservation would mean less diversity preserved in the sediments? How can we know that the original diversity was not even higher? I fail to understand the direct correlation between these two features.
Line 113: Ice Rafted Debris has not yet been introduced as abbreviation
Line 114: change “increases with” with “increases by”
Line 123: change “with higher a sedimentation rate” with “with a higher sedimentation rate”
Lines 120-125: the lack of sufficient resolution in other parts of the core has been commented on several times. I would suggest describing this issue at the beginning of the results chapter or in the sampling section of the Methods, so that you do not need to repeat it for every indicator.
Lines 135-137: Cercozoans were still dominant in the first part of GI8, yes, but it seems to me that after ca. 37.7 ka this dominance is not evident anymore and Apicomplexa abundance is basically the same as Cercozoans.
Line 149: the sea ice free period in the figure is 5-8 ka, why is it 3-8 in the text? Could the time slice from 3 to 5 ka be added to the figure?
Line 150: remove the bracket after Sagenista
Line 153: there is a strong decline in sea ice indicators in GI8, yes, but at the same time there is little evidence of increased open water indicators and this is worth mentioning
Line 171: but this is in the same period when brassicasterol suggests a pretty stable increase in primary productivity, albeit still limited. Your reference here (Griem et al., 2019) refers to foraminifera productivity, but I disagree on the fact that the same is true for the entirety of the primary productivity. Iceberg discharge could also be a source of nutrients, especially iron (e.g., Romero et al., 2021; Barkley et al., 2024), and this would not be the first record showing an increase in primary productivity contemporary to iceberg/sea ice melting
Line 178: here there is still seasonal sea ice though, judging by IP25 contents
Line 186-199: as stated, ecosystem changes seem to have an offset if compared with environmental variables, but at the same time it is interesting to note that, while brassicasterol/productivity rapidly increases at the onset of GI8, the decrease of Cercozoa and the related community shift start later. Do you have any possible explanation for this?
Lines 198-199: change “which in at this time is most likely via subsurface warming” with “most likely via subsurface warming”
Lines 215-216: move “gradually” after “became”
Lines 226-227: that is partially true, but IP25 concentration drops relatively faster than sea ice indicator ASVs (almost 1 ka of offset). This could be due to the uncertainty of the two different age models, but also to a small period when seasonal sea ice was not present and perennial sea ice conditions returned, even though this would not align well with the beginning of the B-A. Either way, it deserves mentioning.

Line 244: the drop in SSTs during MWPs is likely true, due to the relatively fresh and cold surface waters sourced from melting ice sheets, but the alkenones-derived SSTs from You et al. (2023), during MWPs, are not directly related to temperature but on the change of the alkenone source, with an increase of the tetraunsaturated C37:4 coming from freshwater/sea ice communities (when C37:4 is higher than 5%, temperature reconstructions are not reliable (Wang et al., 2021))
Line 254: decide whether to use multi-year or multiyear throughout the text
Line 258: change “for the current and future of Arctic sea ice and the ecosystem it supports” with “for the current and future Arctic sea ice and the ecosystem it supports”
Line 287: a space is missing after “sediments”
Line 303: why calibrating with Marine13 and not Marine20? (Heaton et al., 2020)
Line 305: a bracket is missing at the end of the chapter
Line 307: citation does not have the right format (direct citation)
Line 310: change “collected prior to any other proxy work was initiated, this to ensure that” with “collected prior to any other proxy work was initiated, to ensure that”
Line 324: change “Mili-Q” with “Milli-Q®” or “ultrapure”
Lines 420-422: references are needed to define the open ocean and ice-covered periods
Line 422: change “permanent” with “permanently”
Line 652: change “show” with “shows”
Line 653: Rasmussen et al. should be a direct reference. Change “All plots are on” with “All plots shows”
Line 657: change “records for” with “records from”. The reference for the NGRIP oxygen isotope record is missing
Line 662: change “detritus” with “debris”
Line 663: change “indicating marine productivity” with “indicating overall marine productivity”
Line 668: change “show is” with “shows”
Line 669: Rasmussen et al. should be a direct reference. Change “All plots are on” with “All plots shows”
Line 673: The reference for the NGRIP oxygen isotope record is missing
Line 678: I guess “MW14” should be “MWP1-4”
Line 679: change “indiciated” with “indicated”
Line 680: change “All plots are on” with “All plots shows”
Line 690: change “22CCB” with “22CC-B”

REFERENCES

A.E. Barkley, G. Winckler, C. Recasens, M.R. Kaplan, B.G. Koffman, F. Calabozo, J.L. Middleton, R.F. Anderson, Y. Cai, L. Bolge, J. Longman, & S.L. Goldstein (2024). Patagonian dust, Agulhas Current, and Antarctic ice-rafted debris contributions to the South Atlantic Ocean over the past 150,000 years, Proc. Natl. Acad. Sci. U.S.A. 121 (31) e2402120121. https://doi.org/10.1073/pnas.2402120121

S.T. Belt (2018). Source-specific biomarkers as proxies for Arctic and Antarctic sea ice. Organic Geochemistry, Volume 125: 277-298. https://doi.org/10.1016/j.orggeochem.2018.10.002.

Heaton TJ, Köhler P, Butzin M, et al. Marine20 - The Marine Radiocarbon Age Calibration Curve (0–55,000 cal BP). Radiocarbon. 2020; 62(4): 779-820. doi:10.1017/RDC.2020.68

Romero, O. E., LeVay, L. J., McClymont, E. L., Müller, J., & Cowan, E. A. (2022). Orbital and suborbital-scale variations of productivity and sea surface conditions in the Gulf of Alaska during the past 54,000 years: Impact of iron fertilization by icebergs and meltwater. Paleoceanography and Paleoclimatology, 37, e2021PA004385. https://doi.org/10.1029/2021PA004385

Wang, K.J., Huang, Y., Majaneva, M. et al. Group 2i Isochrysidales produce characteristic alkenones reflecting sea ice distribution. Nat Commun 12, 15 (2021). https://doi.org/10.1038/s41467-020-20187-z

** Visit Nature Portfolio's author and referees' website at www.nature.com/authors for information about policies, services and author benefits**

Open Access This Peer Review File is licensed under a Creative Commons Attribution 4.0 International License, which permits use, sharing, adaptation, distribution and reproduction in any medium or format, as long as you give appropriate credit to the original author(s) and the source, provide a link to the Creative Commons license, and indicate if changes were made.
In cases where reviewers are anonymous, credit should be given to 'Anonymous Referee' and the source.
The images or other third party material in this Peer Review File are included in the article's Creative Commons license, unless indicated otherwise in a credit line to the material. If material is not included in the article's Creative Commons license and your intended use is not permitted by statutory regulation or exceeds the permitted use, you will need to obtain permission directly from the copyright holder.
To view a copy of this license, visit https://creativecommons.org/licenses/by/4.0/

Rebuttal to the reviews

Reviewer #1 (Remarks to the Author):

Dear authors,
The submitted works from De Schepper et al., entitled "Marine community shifts at past rapid climate transitions" consists of original research on an important, yet undocumented effect of Arctic Sea ice cover loss on non-fossilized past biodiversity, using sedimentary ancient DNA (sedaDNA) for over 100 samples taken along a sediment core and extensive glaciology proxies to contextualize changes for micro-eukaryotic taxa that include major planktonic components. These **innovative and robust features all make this work a novel contribution worthy of publication for a broad readership** including oceanographers, paleontologists, ancient molecular biologists, and potentially modelers interested in harnessing omics data to learn from past condition reconstructions. As per my particular expertise on ancient sedimentary DNA, I believe that **the high-quality of this contribution will influence thinking in this field**, with its most-expected application to glacial-deglacial events reconstruction that's critical in the Arctic. Since my expertise lies not in the fields of glaciology or paleontology, my review and comments mainly deal with the sedaDNA molecular methods, bioinformatics and biostatistical analyses of the sequencing data.

To review its structure and assess redundancies, I broke down the manuscript to the paragraph level, each with a summary of the main conveyed message and main comments that I hope will be helpful. In view of this structure breakdown (see below), **it appears that there is redundancy** – especially in the Discussion section – which could be greatly reduced given the efficient and clear writing skills of the authors. Shortening the manuscript by aggregating the salient sedaDNA findings associated with glaciation-deglaciation events more directly and concisely, will but make this flawless work an even more impactful contribution to the field. Indeed, data amount and reported changes seem highly promising so that the authors could offer a revised version with moderate effort.
Answer: We have restructured the manuscript substantially, with a more expanded section describing the results only, and more streamlined discussion where the results are no longer repeated.

One effort yet concerns the statistical analysis, which consist in (i) describing alpha diversity increases/decreases, (ii) identifying community shifts, and beta diversity differentiation events using a suitable moving-window approach, and (iii) providing relative abundances for major taxa between (groups of) samples marking glaciation-deglaciation events. For (ii), more precision would be needed to support claims of "significant community shifts" (a result placed in the Discussion section, but not substantiated in the Results section): what test statistic was measured to claim significance? Also, the methods specify that "matrix was used as input for a Non-Metric Multidimensional Scaling (NMDS) using the metaMDS function", but this analysis is not presented: maybe it would help measuring significance levels?
Answer: We agree with the reviewer that we were unclear regarding significance of shifts. The significance in community shift is assessed via permutation tests in the split moving-window. We added this in the method section (L419) and reported on p-values in the result section (L126). As for the NMDS, we removed this analysis from

the methods, as we here focus on shifts in alpha and beta-diversity over time, i.e. not overall community structure that could be discussed with the results of an NMDS.

For (iii), it is important to consider that sequencing omics data is compositional (doi.org/10.3389/fmicb.2021.727398). This is especially true in this study where the Cercozoa component might include benthic ASVs whereas the diatoms ASVs are likely pelagic. Thus an observed decrease in relative abundance of diatoms might only be due to benthic cercozoans increasing in true abundance in the benthic sample, whereas the true abundance of diatoms would possibly not have changed, or even, increased in surface waters, but possibly not to a point that its sedaDNA would be more abundant than authochotonous benthic cercozoan DNA. This major drawback can be addressed by "considering pairwise ratios of the components and by analyzing these ratios after logarithmic transformation". This is quoted from the above DOI article, which proposed using ALR transform "the essential step being the choice of the reference component". Hence the authors could tackle both my comments by selecting a prevalent enough yet least variable (set of) ASV(s) to use a reference and that would enhance interpretability and highlight this key issue to the field of sedaDNA analysis. For example, strictly pelagic taxa/ASVs could serve as internal references for such logratios calculation and assert the reported changes at glaciation/deglaciation events. Such log-ratios could be computed only for the major events/shifts to provide support to the claims.
Answer: Yes, we fully agree with reviewer that omics datasets are compositional. Also, we agree that the ALR transform could offer benefits and help reduce the statistical issue of compositionally, provided that a solid reference could be identified. However, we think that we cannot use any of the detected ASV in our datasets as reference for transforming the reads count in satisfying manner with ALR transformation. Cercozoan include both planktic and benthic taxa, as mentioned by the reviewer, so it is hard to pick a reference ASV. One may suggest a known benthic taxon (e.g. nematode or a benthic foram for instance), however, we think that it is reasonable to assume that changes in sea ice likely impact both pelagic and benthic communities, making the selection of a reference ASV complicated yet again. With these shortcomings in mind, we do not report or discuss change in "true" abundances in taxa composition, but constantly use "relative" abundances when documenting community variations. Nevertheless, we added this issue as a "disclaimer" in our result section (L82-87), as follow: "Also, it is important to note that our metabarcoding dataset, like any high-throughput sequencing dataset, is compositional (Gloor et al., 2017, 10.3389/fmicb.2017.02224; Greenacre et al., 2021, 10.3389/fmicb.2021.727398), meaning that the microbial load in the sample is unknown, and so the read counts of a given ASV is tightly dependent on all other ASVs of the dataset. We therefore document community shifts in terms of changes in relative abundances in our data."

Introduction
§1 = Sea ice change events in the Arctic and in the Labrador Sea from 50 kyrs to now. Main comment: "Several degrees on matter of decades" is somewhat approximative: could a more accurate number be given?
Answer: we provided more accurate numbers "by 5 to 16˚C within decades"
§2 = Impact on biodiversity and strength of sedaDNA to include the non-fossilized majority into the analysis. Main comments: - It would be good to provide more examples/names of taxa uncovered using sedaDNA. Indeed, "restricted view", "more

complete picture", and "organisms that do not produce a shell, test or cyst" are too broad for the readership to grasp a sense of the large diversity that can be encompassed in sedaDNA analyses. Could be extracted from the cited papers, notably from that focused on the Arctic. It is important for those taxa belonging to phyla well known in the fossil record (e.g., soft-shelled forams) and it would be good if a sense of the large amount of non-fossilized diversity could be given.
Answer: examples with references provided to elaborate on "the more complete view" (L55-59).

§3 = Current influence and sea ice change in the Labrador Sea, incl. major events
Could "Labrador Sea" be labeled on Fig 1?
Answer: Figure 1 has been updated.

Main comments: - There seem to be redundancy with the §1 where is clearly explained that: "the glacial Labrador Sea was characterised by stable, multiyear sea ice conditions during Greenland stadials (GS) whereas during the Greenland interstadials (GI) unstable, seasonally sea ice free conditions occurred", vs here in §3, that "During the Last Glacial, the Labrador Sea was mostly permanently sea ice covered, but during warm interstadials a seasonal sea ice cover occurred, and even open ocean conditions may have occurred during summer". To me this is the same, thus I recommend that the Intro section is slightly revised to introduce once only all the Labrador Sea changes relevant to the study. Also, are the three cited references in §3 all pointing to the same fact, or could there be some additional relevant details to more accurately extract from this literature?
Answer: the introduction was rewritten, and we condensed and removed redundancy from the first paragraphs.

This §3 should provide a brief account of the question addressed more precisely, of the data constituted for this research, of the expected results and extended scope for a broad readership.
- "During the deglaciation […] during the Holocene": as constructed, these durations are not clear, please could approx. times be indicated or the sentence cut into two?
Also, this continued account of key events would best fit before explaining the sedaDNA approach as it seems, is a logical continuation of §1. Again, the **§3 could be more focused on what is unknown** and that could help advance knowledge about past sea ice cover changes, how including more biodiversity can help, and what is done here (and expected) to make such advance.
Answer: As above, the introduction pararagraphs were rewritten

Results The whole section consists of a single paragraph: the first half relates the methods and sedaDNA sequencing yields. The other half superficially presents the taxa, their dominance patterns derived from relative abundances and that diversity shifts were identified that coincide with sea ice cover transition events. It represents a "teaser" for the actual results that are well elaborated in the following Discussion section. Indeed, the brevity of the Results section is surprising as the Discussion section is lengthy and substantiated with many results, (e.g., "During GI8, alpha diversity…"). Hence, I encourage the authors to merge everything into a Result & Discussion section (if agreed with editors), or to elaborate with results sorted off the Discussion.

Answer: the results section (L73-156) was restructured and rewritten. The results sections is now built up as follows: general context of our data, DNA preservation, alpha diversity and beta-diversity patterns, planktic and benthic relative abundances shifts through time, and a new indicator analyses not previously presented. We also moved all results on assemblage shifts that were previously presented in the discussion section, to the results sections.

Either way, slightly more taxonomic details would be useful to identify the ASVs of likely pelagic origins to use in the analyses, as only these ASVs would provide the non-"restricted view on communities and biodiversity of the water column". Re-running the analyses based on a (likely pelagic) subset of the ASVs would represent relatively little work, and provide the sedaDNA community with an highly-relevant, rare comparison.
Answer: We agree with the reviewer that it would be very valuable to be able to analyse separately the response of plankton/benthic communities to sea ice changes. However, making this classification is complicated, as many groups include both planktic and benthic taxa (e.g. Cercozoan mentioned above, but also copepods, ciliates, etc), making such classification based on taxonomic annotation thus problematic. Importantly, we consider that not just plankton communities, but also benthic communities respond to a change in sea ice cover. We have supported this assumption with references from the literature on pelagic-benthic coupling (L80).

Discussion §1 = alpha diversity high (vs low) in deglacial (vs glacial) conditions
Main comments:
- "clearest increase": which statistic based on alpha diversity measures supports this claim?
- the message that "alpha diversity is associated with highly variable ocean conditions, where both sea ice and warm Atlantic waters can occur simultaneously or sequentially" is not very clear. Does it mean that alpha diversity and ocean conditions are correlated?
Answer: we agree that our choice of words was not great here and we have tried to remove subjective qualifying words such as "clearest". We have also tried to be more careful when describing alpha diversity in relation to the ocean conditions (e.g. L107).

§2 = beta diversity, community changes occur at major events where the abundance of major taxa increase/decrease; sampling resolution is an issue but not at HE4. Main comments: - "significant community shifts": which statistic based on beta diversity measures supports this claim? Statistically robust results about the changes in reported taxa abundances are missing in the Results section.
Answer: we agree and have amended the manuscript with details as to how statistical significance was assessed (see also earlier response). We report p-values for the split moving window analysis in the manuscript in the methods (L419).

§3 = alpha, beta and taxa changes along highly resolved GI-GS and water freshening shifts.
- Redundancy of the topic but seemingly contradicting with respect to alpha diversity, between "In the late part of GS9, melting icebergs release IRD to the sediment (HE4), and the surface waters become fresher (Fig. 3d). At peak IRD release, we record a decrease in alpha diversity", and:

(1). “Alpha diversity bounces back rapidly to increase towards the end of the GS9” (§3)
(2). “Consistent high alpha diversity is recorded at the end of GS9, following the melting of icebergs in the Labrador Sea as shown in the IRD record (Figure 3d)” (§1)
- “shift is mostly driven by”: any formal statistical measure?
- Also, it seems essential that the “GI”, “GS”, “HE” periods/events are directly indicated on all plots as for figure 1 (“Greenland Stadials (GS) in black, Greenland Interstadials (GI) in red)”: these are missing form figure 3, making it hard to read alongside the text.
Answer: the rewriting of the discussion and redrafting of the figures has addressed this comment.

Also, please homogenize “Fig.” vs “Figure” notation style.
Answer: done.

§4 = alpha, beta and taxa increase at GI8-GS9 transition as sea ice recedes. Redundancies between “at the transition from GS9 to GI8, we record a decrease in alpha diversity and a community shift when the multi-year sea ice cover is rapidly replaced by seasonal sea ice” and: (1). “In the late part of GS9, melting icebergs release IRD […] At peak IRD release, we record a decrease in alpha diversity and a significant shift” (§3) (2). “Consistent high alpha diversity is recorded at the end of GS9, following the melting of icebergs in the Labrador Sea as shown in the IRD record and the rapid warming at the GS9-GI8 transition” (§1) This is another example of what makes the discussion too verbose, because it scatters the **same messages in multiple paragraphs**. Note that I am not reporting any further instance of redundancy, as this is the authors’ task to carefully revise.
Answer: the results and discussion were rewritten to remove redundancy.

§5 = low alpha diversity and (unspecified) beta changes, and taxa increase at GI8-GS8 transition
Main comment: - “diatoms and Apicomplexa became more abundant at the expense of Cercozoa”: this would necessitate log-ratio comparions between the groups of samples before and after this “becoming”.
Answer: we argued in an earlier response as to why we don’t think log-transforms are useful/feasible. We have also improved our choice of words and have rewritten the text, highlighting consistently that we are reporting relative abundance changes (e.g. L134-138).

§3, §4 and §5 seem to describe in detail a series of events that rapidly succeed one another between 39 and 38 ka b2k (since the resolution is higher for this period), but the level of redundancy amongst them and with §1 is too high.
Answer: the introduction and discussion were rewritten to remove redundancy.

§6 = More recent warm-water ice melting drives increase in alpha and diatoms abundance; alpha high as sea ice recedes.
Main comments: - “This changes during the B-A, when the first of a series of meltwater pulses is recorded”: the meaning of “This changes” is not clear. Is it about the dominance patterns? For all taxa? And to what result does it changes?
Answer: this was rewritten (L139-141)

- High verbosity of “increasing Greenland and Labrador Sea temperatures and decreasing sea ice cover” followed by similar meaning of “seasonal and marginal

sea ice […] gradually becomes more influenced by warmer Atlantic waters". I would suggest shortening to the essential: discussing results.
Answer: this was rewritten (L212-215)

- "increase in diatom ASV abundance": please use compositional data analysis to confirm this or heavily discussion the aforementioned shortcoming about relative abundance issues and the fact that sedaDNA could in fact just have less benthic DNA on the rest of the relative abundance total.
Answer: We acknowledge this issue and have responded above to a similar comment how we deal with this.

§7 = Even more recent warm-water ice melting drives increase in alpha and diatoms abundance; possible change of the benthic community;
Main comments: - "alpha diversity gradually increases (Fig. 4g) with a disappearing sea ice cover and increasing Labrador Sea temperatures as the region becomes more influenced by warmer Atlantic waters". While this is from another time period (yet not clearly bounded as "during deglaciation"), this is yet another similar verbose sentence documeting the same patterns above, i.e., some level of redundancy.
- Also redundant: "The late glacial and early deglacial up to the Bølling-Allerød marine communities were dominated by Cercozoans,
- Also redundant: "The late glacial and early deglacial up to the Bølling-Allerød marine communities were dominated by Cercozoans, Radiolaria and Apicomplexa" and from §6 "Prior to the Bølling-Allerød (B-A), the marine community was dominated by Cercozoans, Radiolaria and Apicomplexa"
- Other sentences are exactly redundant with the rprevious paragraph. My opinion is that such systematic repeats but for different periods would best be presented using a table.
Answer: this was rewritten entirely (L212-253)

- "Here, alpha diversity is highest in our entire record, indicating that warm open waters maintain a rich community" repeats §6 "Here the alpha diversity is highest in our entire record, indicating that warm, sea ice free waters maintain a rich community". In fact, it seems that the authors made a mistake when submitting, a version with duplicated subsections…
Answer: yes – we noticed too that a paragraph was duplicated in the submitted version. In this submission, the entire discussion was rewritten.

§8 = reflection on todays' changes and the strenght of sedaDNA
Main comment: owing to my main comment about the superficiality of the results at high-level taxa and the lack of scrutiny to distinguish benthjic vs "surface ocean biosphere" taxa makes the following claim an overstatement: "We documented the effect of climate change and a changing sea ice environment on the surface ocean biosphere, and our metabarcoding study firstly demonstrates that DNA stored in Late Quaternary marine sediments can document past community changes."
Answer: this statement is no longer in the new text, as the entire discussion was rewritten.

Moreover, the fact that non-fossilized taxa that 18S primer will recover is not a novel demonstration.
Answer: we agree and have changed the text

§9 = highlights of main events translated into diversity changes and their frequency
No comment as this is the key relevant and strength of this work and should be the focus of more elabiorate result and impactful discussion, that could also cite previous sedaDNA results for other events/changes.
Answer: We are unsure what the reviewer is referring to here, but since the discussion section was entirely rewritten, we believe this was addressed.

§10 = the global impact of diatom surges is ice free waters
Main comment: I feel that this last paragraph is too verbose to convey the intuitive activation of biological pumps in ice free conditions, and thus loses the focus on community-wide shifts (i.e., the title). I would even suggest reducing text about this mechanisms to two sentences dealing with the Southern Ocean example on the importance of a taxon like diatoms to model deglaciation impacts on atmCO2 and moving them in the Introduction section. Thus, the Discussion section could more briefly reflect the diatom increase results onto this mechanism, ot then speculate and invite for further high-resultion sedaDNA research on climate feedback at glacialdegracial interfaces.
- “a shift to a diatom-dominated ecosystem shift” is not clear. Quite clearly, the main messages is of higher/increased alpha diversity and beta diversity shifts when sea ice recedes. Because it is scattered across multiple paragraphs, this trend is more difficult to grasp. Hence, my suggestion of increasing the results section with tests to better assert these changes, and of reducing the discussion to explain this trend once, using all the different sea ice change events as systematic, concurring evidence.
Answer: we have rewritten and refocused this section and included references to new literature (e.g. Ramondenc et al 2025, Weiß et al. 2025). The text (L233–253) now focuses on the links between the meltwater pulses, the shifts in marine communities, and the different sea ice conditions during MWP1 vs MWP2-4. In L255–283, we highlight how cryosphere and marine communities are linked in the light of ongoing sea ice change in the Arctic, and suggest a future direction for paleoclimate studies to improve our understanding of ecosystem changes impact climate during climate warming events.

Reviewer #2

De Schepper and co-authors are presenting sedaDNA results for glacial to Holocene sections of a sediment core recovered off the southern tip of Greenland. Those are the first results for that region focusing on the eukaryotes in general during those particular periods. The authors observe clear changes in the plankton (?) ecosystem composition along with the different background climate scenarios and indicate that their results could provide insights into the future evolution of the Arctic Ocean. The results merit publication, but the current manuscript is in no shape to be published and, in my opinion, does the data not justice. I was very exited reading the manuscript title and the abstract, but then ended up underwhelmed when reading the discussion, which is very descriptive and short on “ecosystem scenarios”. The manuscript is doing a poor job bridging between the different disciplines leaving the reader a bit lost. For example, with my background in paleoceanography some of the eukaryotes groups mentioned are unknowns and the authors fail to mention if all

those groups are plankton, what size plankton, an important information to know where in the ecosystem functioning food chain they are relevant, or if they are common in the modern subpolar/polar ocean.

Answer: While using a high-level taxonomy is not uncommon for metabarcode datasets, we acknowledge that such high-level taxonomy may not be immediately clear to the reader who is more accustmed taxonomy used in e.g. paleoceanography (e.g. diatom genera, foraminifer species). However, our approach with metabarcoding does not allow (with some exceptions) to go to genus/species level. It is often not possible to assign our taxonomic groups to plankton or benthos (i.e. Cercozoa are known from soils, fresh water and marine systems). However, we have given some more ecological info where possible on e.g. Apicomplexa (parasitic) and Cercozoa (heterotrophic) to help the reader.
With respect to the modern distribution of our taxonomic groups, see our response below.

For someone working in marine biology/ecosystem function, the description "the ecoystem changes" would probably not be enough as I would like to know in what direction/to which ecoregion etc. (and thus potentially what such a change could mean for the ecosystem in general, including fishes and mammals).
Answer: we have rewritten our results and discussion section and, where relevant, have highlighted whether the marine communities are diatom or parasite dominated (L148, 188, L277). We have been more careful when writing about our data, which is showing community rather than ecosystem changes. We don't have any information on how fish or mammals are affected from our dataset, so we could only speculate. While that would be interesting, an entire paleo-ecosystem reconstruction is beyond the scope of our paper.

In this context, I am missing any comparison with Tara Ocean genomics results (or others) that could help to describe if the encountered eukaryote groups are typical for the Labrador Sea, the Nordic Seas or the Arctic Ocean, so that observed past ecosystem (well, in reality only a small part of the ecosystem) changes could be related to present day oceanographic conditions.
Answer: We agree that comparing our data to a modern dataset would be valuable, if such dataset has a significant number of samples in our study area, is based on seafloor surface sediments and plankton samples, and targeting the same marker (V7 in our case). Neither exist at the moment. The Tara Oceans dataset from the water column is based on V9 and V4 and has few relevant stations for our study area. Only 1 station has genomic data from the Labrador Sea (210). We find a n=1 dataset would not provide a meaningful comparison. Looking further in subpolar areas, the Baffin Bay sites are mainly coastal sites (206, 208, 209) and represent a different environment than our open ocean location. There are two stations in the Norwegian Sea (158, 163) which track the warm Atlantic water current, which is again a setting not comparable to the Labrador Sea. All polar sites are from shallower, shelf settings, which we don't consider to be relevant for comparison. Therefore, we consider a comparison to a modern dataset such at Tara Oceans in our context (not the same marker) to be misguided and also beyond the scope of this study. Here, we focus on past eukaryotes communities and the changes these have undergone in relation to the environment in the past.

I was also hoping for a better integration with existing palaeoecology data from the region, such as the **planktonic foraminifer abundance data from the Griem et al. study** at the same location or microfossil evidence from the wider region, especially **regarding the diatom abundance peaks during the last glacial/interglacial transition**.
Answer: The Griem et al. dataset (25-65 ka b2k) does not cover the last deglaciation. It overlaps with a part of our studied interval (5-45 kyr b2k), and where relevant (~38 ka b2k) we have included their abundance data and IRD data (e.g. Figure 3b,c,d). When we refer to Griem et al. (2019) in the discussion, we use their foram data to provide the paleoceanographic context (e.g. L172,180,185,192, 200, etc.).

I put a related comment and potential publications to look at in a comment in the uploaded annotated manuscript. For example **have indicators for higher productivity of diatoms counterparts in increased production in zooplankton?** The recently published sedaDNA study (Grant et al., 2024; by the same group) for the previous interglacial period and preceding glacial in the same sediment core is doing a much better job in that perspective; a study, which is not even mentioned in the current manuscript.
Answer: We did not act on this comment and the reference list of the reviewer, as this is beyond the scope of our paper. We were not looking for indicators, or diatom-zooplankton links; rather, we intended to highlight that past communities undergo changes in relation to the sea ice conditions.
The publication by Grant was published after our previous submission, hence it was not included then. It is now included as a reference.

Here are some modern ocean references, which could help to improve the manuscript (although I did not check in detail): Pierella Karlusich, J. J., F. M. Ibarbalz, and C. Bowler (2020), Phytoplankton in the Tara Ocean, Annual Review of Marine Science, 12(1), 233-265, doi: 10.1146/annurev-marine-010419-010706. Ibarbalz, F. M., et al. (2019), Global Trends in Marine Plankton Diversity across Kingdoms of Life, Cell, 179(5), 1084-1097.e1021, doi: https://doi.org/10.1016/j.cell.2019.10.008. Righetti, D., M. Vogt, N. Gruber, A. Psomas, and N. E. Zimmermann (2019), Global pattern of phytoplankton diversity driven by temperature and environmental variability, Science Advances, 5(5), eaau6253, doi: 10.1126/sciadv.aau6253. Richter, D. J., et al. (2022), Genomic evidence for global ocean plankton biogeography shaped by large-scale current systems, eLife, 11, doi: 10.7554/eLife.78129. Arrigo, K. R. (2014), Sea Ice Ecosystems, Annual Review of Marine Science, 6(1), 439-467, doi: 10.1146/annurev-marine-010213- 135103. Fragoso, G. M., A. J. Poulton, I. M. Yashayaev, E. J. H. Head, M. C. Stinchcombe, and D. A. Purdie (2016), Biogeographical patterns and environmental controls of phytoplankton communities from contrasting hydrographical zones of the Labrador Sea, Progress in Oceanography, 141, 212-226. Fragoso, G. M., A. J. Poulton, I. M. Yashayaev, E. J. H. Head, G. Johnsen, and D. A. Purdie (2018), Diatom Biogeography From the Labrador Sea Revealed Through a Trait-Based Approach, Frontiers in Marine Science, 5, doi: 10.3389/fmars.2018.00297. Mayot, N., P. A. Matrai, A. Arjona, S. Bélanger, C. Marchese, T. Jaegler, M. Ardyna, and M. Steele (2020), Springtime

Export of Arctic Sea Ice Influences Phytoplankton Production in the Greenland Sea, Journal of Geophysical Research: Oceans, 125(3), e2019JC015799.

Marine community shifts at past rapid climate transitions

Stijn De Schepper[1], Tristan Cordier[1], Katrine Sandnes Skaar[2], Jessica Louise Ray[2], Kristine Steinsland[1], Danielle M. Grant[1*], Henrik Sadatzki[3], Bjørg Risebrobakken[1] and Aud Larsen[2]

[1] NORCE Norwegian Research Centre & Bjerknes Centre for Climate Research, Bergen, Norway
[2] NORCE Norwegian Research Centre, Bergen, Norway
[3] MARUM – Center for Marine Environmental Sciences, University of Bremen, Bremen, Germany
*Current address: Hakai Institute

ABSTRACT

Today, sea ice cover is rapidly declining in the Arctic Ocean and major changes in the Arctic marine ecosystems are starting to appear. The full implication of a changing Arctic ecosystem is not well understood as past time series across rapid climate events are scarce, and usually focused on a single group of organisms (i.e. foraminifers). A more complete, holistic view on the marine communities can be obtained via analysing ancient DNA metabarcodes of marine eukaryotes stored in marine sediments. Here we demonstrate that major community shifts were associated with abrupt climate warming events over the last ~50,000 years in the Labrador Sea. Shifts happen when sea ice conditions changed and meltwater from icebergs or a melting ice sheet enter the ocean. During the last deglaciation, marine communities became dominated by diatoms and this likely increased export production and biological pump efficiency and dampened the deglacial CO_2 increase. Large community shifts thus happen when sea ice disappears and may have contributed to the deglacial climate evolution.

INTRODUCTION

Over the last 50,000 years of Earth's history, global climate changed from cold, glacial conditions to the warm, interglacial conditions we experience today. During most of the last glacial, near perennial sea ice covered the northern North Atlantic . Between ca. 50,000 and 25,000 years ago, the glacial Labrador Sea was characterised by stable, multiyear sea ice conditions during Greenland stadials (GS) whereas during the Greenland interstadials (GI) unstable, seasonally sea ice free conditions occurred[1–3]. On Greenland, temperature increased with several degrees over a matter of decades during stadial to interstadial transitions[4] and in the northern North Atlantic the permanent sea ice cover was replaced by seasonal sea ice to open water conditions[2,5]. These events are therefore very relevant in the light of the ongoing and projected sea ice change in the Arctic[6,7]: here a transition from a sea ice covered ("white") to a sea ice free ("blue") ocean is ongoing, and is making an imprint on Arctic Ocean biodiversity already today[8,9], and this will only continue in the future[10,11].

While the mechanisms underlying rapid climate change events in the glacial ocean have been investigated[4,12], there is little known about the impact of these events on marine biodiversity. Traditionally, paleoceanographic studies use microfossils, as these are widespread and often abundantly present in marine sediments. However, in sea-ice-covered oceans, calcareous and

siliceous microfossils are often poorly preserved and show low diversity[13]. Additionally, microfossil studies often focus only on a single group of organisms, providing a restricted view on the communities and biodiversity of the water column. An approach that uses marine sedimentary ancient DNA (*seda*DNA) can provide a more complete picture of past biodiversity[14–17] compared to single-organism microfossil studies[18]. DNA stored in sediments does not only include known organisms from the fossil record (foraminifers, diatoms, dinoflagellate cysts), but also organisms that do not produce a shell, test or cyst during their life cycle (e.g. cercozoa).

Today, the Labrador Sea is influenced by warm Atlantic water from the Irminger Current and cool, polar water from the East Greenland Current (***Fig. 1***). During the Last Glacial, the Labrador Sea was mostly permanently sea ice covered, but during warm interstadials a seasonal sea ice cover occurred, and even open ocean conditions may have occurred during summer[1,2,19]. Two major Greenland-sourced iceberg calving and melt water events were recorded at our study site[1]: Heinrich Event (HE) 4 in GS9 and HE3 in GS5.1. Several stadials and interstadials (GS9–GI8; GS5.1–GS4) are well expressed in the foraminiferal records at our study site, but others are not (e.g. GI7 to GI5.1) (***Fig. 2b***). During the deglaciation, the permanent sea ice was replaced with seasonal sea ice and several meltwater pulses are recorded in the Labrador Sea before entering stable open ocean, modern-like conditions during the Holocene[19,20].

RESULTS

Here we present a Late Quaternary sedimentary ancient DNA (sedaDNA) record that documents past marine communities back to ca. 50,000 years ago in a sediment core from the Labrador Sea (GS16-204-22CC-B, 58° 02.83 N, 47° 02.36 W, water depth 3160 m; Fig. 1). We extracted DNA from 114 sediment samples, followed by PCR-amplification and sequencing of the V7 hypervariable region of the eukaryotic small subunit ribosomal RNA (18S SSU rRNA) on an Illumina MiSeq platform. After standard quality filtering steps of the raw sequencing data (see Supplementary Information/Methods), we taxonomically annotated the Amplicon Sequence Variants (ASVs, Callahan et al., 2016) using the PR2 reference sequences database[21] and obtained 22,018,189 reads, which grouped into 4,836 ASVs. The ASVs belong to fossilising protists such as diatoms and dinoflagellates, but also to taxa that do not leave behind fossilisable (tests, cysts, shells) remains such as cercozoans, apicomplexans or fungi. We found that eukaryotic communities were dominated by cercozoans (31.5% of the reads), apicomplexans (16.8%) and diatoms (12.8%), while 3.8% of the reads could not be taxonomically annotated. Richness and relative abundances of eukaryotic groups shifted throughout the record (Fig. 2c,d): cercozoans were dominant from 44–41 ky, 32–29 ky, and during 19–15 ky, while fungi, dinoflagellates and opalozoan dominated during 36–32 ky~~,~~ 29–21 ky. The deglaciation is marked ~~with a~~ three pulses of diatom dominance. The analysis of beta-diversity variation, us~~ed~~ a split moving-window analysis of ecological differentiation[22] along the sediment core, revealed a total of seven significant shifts in eukaryotes composition (Fig. 2e). Comparison of our *seda*DNA data to ocean and climate proxy records shows that major changes in taxonomic diversity and shifts in dominant groups occur around rapid climate shifts, and are specifically clearly expressed around HE4, stadial-interstadial transition GS9-GI8, and during the deglaciation when rapid changes in Labrador Sea oceanography occurred.

DISCUSSION

Change in sea ice regime triggers changes in marine diversity

Our analyses of the eukaryotic communities based on *sed*aDNA show a general pattern of low alpha diversity (richness) when the Labrador Sea is covered with multiyear sea-ice, and high diversity when open waters or seasonal sea ice occur. Between ca. 50,000 and 15,000 (b2k) alpha-diversity is low and mostly stable (Fig. 2d), consistent with a multiyear sea ice cover and generally low productivity in the Labrador Sea[1,23,24]. Consistent high alpha diversity is recorded at the end of GS9, following the melting of icebergs in the Labrador Sea as shown in the IRD record (Figure 3d) and the rapid warming at the GS9-GI8 transition. During GI8, the study site was likely located in a polynya environment, or possibly in Atlantic-water-influenced, high-productivity open waters[1,2]. The clearest increase in alpha-diversity occurred during the deglaciation, when global climate warms and seasonal sea ice cover and several abrupt meltwater pulses are recorded in the Labrador Sea[19]. This suggests that highest alpha diversity is associated with highly variable ocean conditions, where both sea ice and warm Atlantic waters can occur simultaneously or sequentially.

Shifts in eukaryote community composition (beta diversity) are identified throughout the studied record. Even though our sampling resolution is not high enough to capture all stadial/interstadial events, significant community shifts are identified around GI6 to GI5.2, where chlorophytes, diatoms and cercozoans increase in abundance, and around GS5.1 to GI4 (HE3), where cercozoans disappear from the record and apicomplexa, opalozoa and fungi become more dominant. These events may align with rapid climate events, but our sampling resolution is too low to confidently conclude this. The most pronounced shift, i.e. highest differentiation index values, occur during Heinrich Event HE4 and the subsequent abrupt climate warming from GS9 to GI8 and (Fig. 2e), and during the deglaciation in the Bølling-Allerød interval (Fig. 2e, 3). Here, our sampling interval is highly-resolved and we discuss both climate transitions and comunity shifts in more detail below.

Iceberg melt and sea ice retreat causes community shifts

Across HE4 (~39 ka b2k) and the transition from stadial GS9 to interstadial GI8 (38–38.5 kyr b2k), the interval between samples is 1.5 cm. This allows detailed and direct comparison of the surface ocean community during this rapid climate transition. During GS9, cercozoans are the dominant group (Fig. 3f) when the site is underlying multi-year sea ice, close to the polar front (Fig. 3c, 3e; Griem et al. 2019; Scoto et al. 2022). In the late part of GS9, melting icebergs release IRD to the sediment (HE4), and the surface waters become fresher (Fig. 3b, 3d; Griem et al. 2019). At peak IRD release, we record a decrease in alpha diversity and a significant shift in community composition (Fig. 3g). This shift is mostly driven by a large change within Cercozoan assemblages, with an increase of 17% of Cryomonadida relative abundance, and a decrease of 6.5% in Ebriida and 10% in an unclassified group of Cercozoan. Alpha diversity bounces back rapidly to increase towards the end of the GS9.

Subsequently, at the transition from GS9 to GI8, we record a decrease in alpha diversity and a community shift (Figs. 3g,f) when the multi-year sea ice cover is rapidly replaced by seasonal sea ice (Fig. 3e) and Greenland experiences increased atmospheric warming (Fig. 3a). This shift is mostly driven by an increase in the relative abundance of apicomplexan assemblages (Neogregarinorida, 7.5%, Agamococcidiorida 6.2% and Eugregarinorida 3.8%) and in Polar-

centric Mediophyceae taxa (6.3%), at the expense of Cercozoan assemblages (Ebriida: 11.7% and Cryomonadida 12.5%).

During GI8, alpha diversity remained low and the sea ice edge retreated and re-advanced over the site (Fig. 3c,e,g), which temporarily may have been positioned south of the Arctic Front and influenced by Atlantic water (Griem et al. 2022, Scoto et al. 2022). Here, diatoms (reaching 22.5% in relative abundance) and Apicomplexa became more abundant (reaching 42% in relative abundance) at the expense of Cercozoa. A change in ASV composition happened again at the transition to GS8 (Fig. 2c) when the site became again covered with near-perennial sea ice.

The rise of diatoms during deglacial meltwater pulses

From around 18–19 ka, seasonal and marginal sea ice conditions occur in the Labrador Sea, which gradually becomes more influenced by warmer Atlantic waters[19,23]. Alpha diversity increases (Fig. 2d, 4g) in this period, in sync with increasing Greenland and Labrador Sea temperatures and decreasing sea ice cover[19] (Fig. 4c,d,e). Prior to the Bølling-Allerød (B-A), the marine community was dominated by Cercozoans, Radiolaria and Apicomplexa. This changes during the B-A, when the first of a series of meltwater pulses is recorded[19]. These meltwater events are characterised by an abrupt decrease in SSTs, increased sea ice formation, and oligotrophic conditions due to strong meltwater discharge. Each meltwater pulse correlates with an increase in diatom ASV abundance. MWP-1 is likely caused by melting icebergs, a scenario comparable to HE4. Interestingly, this is the only melt water pulse that caused a significant shift in communities (Fig. 4h). The latter three pulses (MWP-2, MWP-3, MWP-4) show evidence for suspension-rich meltwater that is likely sourced from the disintegrating Laurentide and Greenland Ice sheets[19]. Between 8 and 5 ka, the Labrador Sea is sea ice free with temperatures just above modern annual temperatures (around 6-8 ˚C) and has elevated productivity[19]. Here the alpha diversity is highest in our entire record, indicating that warm, sea ice free waters maintain a rich community.

During the deglaciation, alpha diversity gradually increases (Fig. 4g) with a disappearing sea ice cover and increasing Labrador Sea temperatures as the region becomes more influenced by warmer Atlantic waters[19,23] (Fig. 4c,d,e). The late glacial and early deglacial up to the Bølling-Allerød marine communities were dominated by Cercozoans, Radiolaria and Apicomplexa. This changed during the rapid warming event of the ~~Bølling-Allerød (~~B-A~~)~~. Here we record a clear community shift (Fig. 4h) and four subsequent peaks in diatom ASV abundance between ca. 14–8 kyr. Each peak in diatom ASV abundance can be directly related to meltwater events in Labrador Sea. The first event, MWP-1, where diatoms ASV abundance peaks for the first time, and the marine community significantly changes (Fig. 4h). All meltwater events are characterised by an abrupt decrease in SSTs, increased sea ice formation, and oligotrophic conditions due to strong meltwater discharge[19]. While meltwater pulses MWP-2, MWP-3 and MWP-4 are characterised by suspension-rich meltwater sourced from the disintegrating Laurentide and Greenland ice sheets, MWP-1 is likely caused by melting icebergs. The MWP-1 scenario thus seems comparable to HE4 (GS9), where also we also recorded a significant community shift when IRD is recorded in the sediment, and icebergs melted near the site. We assume that iceberg meltwater can sustain a different and/or more diverse surface ocean marine community (diatoms), or possibly icebergs and their sediments bring an exotic community to the seafloor at our sites. Meltwater from the

disintegrating Laurentide and Greenland Ice caused three subsequent diatom ASV abundance peaks, with the last peak around 8 ka. Between 8 and 5 ka, the Labrador Sea became sea ice free, was a bit warmer than the modern annual average (around 6-8 ˚C) and showed elevated productivity[19]. Here, alpha diversity is highest in our entire record, indicating that warm open waters maintain a rich community.

Marine community shifts and climate change
In the modern Arctic, multi-year sea ice is disappearing, sea ice extent is rapidly declining, and the Greenland Ice Sheet is shrinking at unprecedented pace. Such change is leading to changes in the ecosystem, yet the rate, magnitude and impact of these shifts are unclear. Our study focuses on Late Quaternary time intervals which show rapid changes from permanent to (seasonally) sea ice free Labrador Sea, a scenario not unlike what is happening in our modern Arctic. We documented the effect of climate change and a changing sea ice environment on the surface ocean biosphere, and our metabarcoding study firstly demonstrates that DNA stored in Late Quaternary marine sediments can document past community changes. Using metabarcoding, we provide a much more comprehensive view of the entire marine ecosystem compared to single group microfossil analyses and we identified several groups of organisms that were previously unknown from the fossil record in the Labrador Sea (e.g. Cercozoa).

Secondly, we show that changes in alpha diversity and community shifts (beta diversity) occurred together with abrupt climate warming events over the last ca. 50,000 years. Our sampling resolution and likely also site-specific conditions did not cover and/or record all rapid climate shifts of the Late Quaternary. Nevertheless, well-documented and significant community shifts occurred during GS9-GI8 (HE4) and the B-A. For both abrupt climate events, the surface ocean ecosystem composition changed substantially and in sync with iceberg melting and the disappearing multi-year sea ice cover. Our data shows that when a sea ice covered ocean turns blue, a fundamentally different ecosystem is installed at the same time. It highlights that marine community shifts occur together with decadal-scale climate shifts, and in view of what is happening in our modern Arctic Ocean, a substantial change to the Arctic marine communities and ecosystems can occur at a comparable rate to the sea ice retreat.

Finally, we speculate that a shift to a diatom-dominated ecosystem shift when a permanent sea ice cover disintegrates may have affected global climate during the deglaciation. Our Labrador Sea data shows a rapid increase in the diatom abundance with each abrupt meltwater pulse during the late deglaciation. A community shift towards diatom dominance affects the benthic-pelagic coupling[25] and can considerably enhance the efficiency of the biological carbon pump, because diatom-derived material degrades slow and can withstand microbial degradation[26]. In this regard, it is interesting to note that a shift to a diatom-dominated ecosystem occurred around 14 ka in the Southern Ocean as sea ice disappeared there[16]. Fogwill et al.[27] proposed that sea-ice biological feedbacks in the Southern Ocean are responsible for the plateau in global deglacial increase in atmospheric CO_2. We speculate that the cause of the plateau is a shift towards diatom-dominated (sub)polar ecosystems, since this would lead to a more efficient biological pump and contribution to dampening of the deglacial atmospheric CO_2 increase. In summary, the Southern Ocean and Labrador Sea marine ecosystem shifts appear contemporaneous and we conclude that marine ecosystem

shifts provide an important feedback to the climate system when a sea ice cover disappeared during climate warming events in the Late Quaternary.

ONLINE METHODS

Materials. During an expedition with RV G.O. Sars in 2016, two calypso cores were taken at Eirik Drift Station GS16-204-22CC (58° 02.83 N, 47° 02.36 W, water depth 3160 m). GS16-204-22CC-A (hereafter 22CCA) and GS16-204-22CC-B (hereafter 22CC-B) recovered Holocene to Marine Isotope Stage 6 sediments[1,28]. For this study, we investigated the MIS 3 to middle Holocene interval between 465 and 70 cm depth of 22CC-B. This sediment core was frozen immediately after splitting the core onboard and kept frozen until sampling in our labs.

Age model. The age model for core 22CC-B is established as follows: we first correlated the colour reflectance parameters a* of 22CC-B with 22CC-A (Fig. S1, Suppl. Table S1). The correlation is confirmed independently around HE4, where 22CC-A[1] and 22CC-B (this study) IRD data overlap (Fig. S2, Suppl. Table S2). Subsequently, we transferred the 22CC-A age model of Griem et al.[1] to 22CC-B. Their age model is based on the correlation of the planktonic foraminiferal $\delta^{18}O$ and $\delta^{13}C$ records to PS2644-5[29], supported by AMS ^{14}C dates, tephra-layers and geomagnetic data. To get a better age control in the Holocene, we added four more AMS ^{14}C dates in the upper part of the 22CC-B, an interval not covered by the Griem et al. (2019) age model. Those dates were measured at Beta Analytics on the planktic foraminifer *N. pachyderma* sinistral and calibrated with Marine13[30], no additional reservoir correction, and added 50 years to get to b2k ages (as for the age model of ref. [1]). Sedimentation rate and age between 0 and 65 cm were linearly extrapolated. The sedimentation rate was higher during the Holocene compared to the last glacial.

Sampling. The sampling for *sed*aDNA analyses was guided by the preliminary work of ref. [2]. We sampled at a higher resolution around the Heinrich Events, initially based on correlation of magnetic susceptibility records of both 22CC-A to 22CC-B. Samples for DNA analyses were collected prior to any other proxy work was initiated, this to ensure that the samples were as pristine as possible for DNA work, where contamination can be a real issue. The 22CC-B core was split onboard during the cruise, covered with plastic film, put in sealed storage tubes and transferred immediately to the freezer. The entire sections were kept frozen (-20˚C) until onshore sampling. Sampling took place in a lab, where the bench space was cleaned thoroughly with bleach (10%) prior to sampling. The sections were gently thawed overnight (at 4 °C) before sampling. Samples were collected using sterile syringes (with cut-off tips) after the upper 2–5 mm of sediment was removed using sterile spatulas. Syringes filled with sediment were put into individual plastic bags and frozen again at -20°C until DNA extraction. All frozen sediments for *sed*aDNA analysis were handled in an access-restricted, positively pressurised room (HEPA filters) intended for nucleic acid extraction, while researchers wore appropriate personal protection equipments (single-use microfibre suits, particle masks, safety goggles and gloves). Room and equipment were thoroughly cleaned with a freshly-prepared 10% (v/v) solution of sodium hypochlorite in Mili-Q water or DNAZap (Thermo Fisher Scientific). Sediment subsampling was done inside a class II laminar flow safety cabinet, whose internal surfaces were decontaminated using DNAZap (Invitrogen™ AM9890) and UV light. Prior to sampling, sediments were thawed at 4°C for 1–2 hours. The plunger was used

to push the sediment out of the syringe, and we removed and discarded the 1 cm of exposed sediment using a sterile scalpel. Six individual subsamples of approximately 0.5 g (wet weight) were weighed directly into DNeasy PowerBead tubes (QIAGEN PowerSoil kit, Hilden, Germany) and frozen at -20˚C until DNA extraction with the DNeasy PowerSoil mini kit (QIAGEN) according to manufacturer instructions. All laboratory equipment and surfaces were cleaned again prior to processing the next sample.

DNA extraction was conducted in the same handling room and safety cabinet where subsamples were taken. Bead-assisted mechanical lysis was performed in a Tissue Lyzer II (Precellys, France) using the following program: 6000 rpm for 40 seconds, 2 min pause, 6000 rpm for 40 seconds, 2 min pause and 6000 rpm for 40 seconds. After purification, DNA was eluted in 100 µl kit elution buffer, divided into two aliquots of 50 µl and stored at -20˚C. One aliquot was maintained as archive, while the other aliquot was utilised as the working DNA stock and always handled inside a clean class II laminar flow safety cabinet.

To identify potential modern DNA contaminants in *sed*aDNA preparations, we performed both sampling and extraction controls. For sampling controls, PowerBead tubes (Axygen, Corning Life Sciences, New York, USA) were opened and placed in a tube rack inside the safety cabinet and left open for random time intervals during sediment subsampling (times were noted). These sampling controls were processed the same as sediment-containing samples for DNA extraction and PCR amplification. For extraction controls, PowerBead tubes containing no sediment material were run through the DNA extraction protocol exactly the same as sediment samples. Eluates from DNA extraction for both sampling and extraction controls were stored at -20˚C until PCR amplification.

We PCR-amplified the V7 hypervariable region of the small subunit ribosomal RNA (SSU rRNA) gene was using universal eukaryote primers (ref. [31]). Sequencing libraries were prepared using a two-step PCR protocol. The first PCR targeted the V7 SSU rRNA gene region using primers F1183mod (5' - AATTTGACTCAACRCGGG - 3') and R1443mod (5'- GRGCATCACAGACCTG - 3') that were appended with Illumina adapters and 12N diversity stretches, as previously described[15]. Fifty microliter PCR reactions consisted of 1U of Phusion® High Fidelity Polymerase (Thermo Fisher Scientific), 1X HF buffer (Thermo Fisher Scientific), 250 nM final concentration of each primer, 0.2 mM final concentration of each dNTP, 0.2 mg/ml molecular biology grade Bovine Serum Albumin (New England Biolabs, USA), 5 µL template DNA, and ultrapure water. The amplification program consisted of an initial denaturation at 95˚C for 5 min, followed by 30 cycles of 95˚C for 20 sec, 59 ˚C for 30 sec, 72˚C for 30 sec, and a final elongation at 72˚C for 10 min. Amplification success and amplicon size (expected to be ranging from 260 to 360 bp) were determined by 1.5% (w/v) agarose gel electrophoresis with 1X GelRed® (Biotium, USA) staining. Amplicons were purified with magnetic beads (MagBio, USA) using a product:bead volumetric ratio of 1.8 in the first round, followed by a second round of purification using a volumetric ratio of 0.8. Purified amplicon concentration was measured with a Qubit fluorometer (Thermo Fisher Scientific, USA).

The second PCR ligated unique combinations of multiplex identifiers (8 unique forward primers (D501-D508) and 12 unique reverse primers (D701-D712)) and Illumina sequencing primer sequences onto the purified amplicons from the first PCR. Fifty-microliter PCR reactions consisted of 1U Phusion® HF Polymerase, 1X HF buffer, 0.2 mM final concentration

of each dNTP, 5 µL template (diluted to 107 purified amplicons from the first PCR) and ultrapure water to 50 µL reaction volume. Each reaction had a unique combination of barcode forward (D5) and reverse (D7) primers, with concentration of 10 µM for forward and 5 µM for reverse. The amplification program followed: initial denaturation at 95˚C for 5 min; 15 cycles of 95˚C for 20 sec, 62 ˚C for 30 sec,72˚C for 30 sec; a final elongation at 72˚C for 10 min. PCR products were visualised by agarose gel electrophoresis and purified with two rounds of magnetic beads using a product: bead ratio of 0.8 for both rounds. PCR products with visible residual primers, or primer dimers, were purified a third time. The concentration of purified amplicons was measured with Qubit and dual-indexed amplicons were pooled in equimolar ratios into a single sequencing library. The library was then purified twice using magnetic beads, first with a volumetric ratio of 0.8 then with a ratio of 1. Purified libraries were visualised using agarose gel electrophoresis and quantified by Qubit and NanoDrop One. The libraries were sequenced at the Norwegian Sequencing Center at the University of Oslo, Norway on an Illumina MiSeq platform using v.3 chemistry and 600 cycles (300 bp x 2). Demultiplexed sequence data was transferred to the Norwegian e-Infrastructure for Life Sciences and archived to the StoreBioInfo repository of the Norwegian Bioinformatics Platform (NorStore/NIRD). Sequence data and sample metadata will be made publicly available on the European Nucleotide Archive repository as BioProject XXXXXXX.

Bioinformatics

We first trimmed the primers sequences from the raw sequencing data using the cutadapt v3.4 software[32] and discarded any read that would not contain both the forward and reverse primers (no mismatch allowed). We then used the DADA2 v1.6 R package[33] with default settings to filter poor quality reads (*filterAndTrim* function), the training of errors models (*learnErrors* function), the inference of Amplicon Sequence Variants (ASVs) with the *dada* function, the merging for paired-end reads (*mergePairs* function), the generation of the ASVs-to-sample matrix (*makeSequenceTable* function) and the filtering of chimeric ASVs (*removeBimera* function). We then taxonomically annotated the ASVs using the PR2 database[21] and the assignment-fasta-vsearch module of the SLIM application[34] that wrap the vsearch toolkit[35]. ASVs were annotated using a Last Common Ancestor approach, i.e. to the consensual taxonomic rank among up to three candidates reference sequences matching the query by at least 85% similarity. ASVs with 99% similarity or more with a reference sequence were directly annotated to that reference. We finally used the decontam R package[36], with default settings, to identify and discard potential contaminants ASVs, by comparing their prevalence between in samples and negative controls (sampling and extraction controls).

We used the vegan v2.6-4 R package[37] to calculate both alpha and beta diversity metrics. For alpha diversity, we calculated the Shannon index and the normalised richness (observed number of ASVs) by subsampling an even number of reads for each sample across the ASV matrix. We then normalised the ASV matrix using the Cumulative Sum Scaling method implemented in the metagenomeSeq v1.4 R package[38], and computed a pairwise Bray-Curtis dissimilarity matrix. This matrix was used as input for a Non-Metric Multidimensional Scaling (NMDS) using the *metaMDS* function. We also performed a split moving-window analysis[22] using the *smwda* function of the EcolUtils v0.1 R packages[39] with default settings, to identify significant compositional shifts along the sediment record.

ACKNOWLEDGEMENTS

We like to acknowledge the invaluable help from our research technicians Dag Inge Blindheim, Katja Häkli, Sigrid Mugu for their help with collecting samples and lab analysis. The crew and scientific party of the ice2ice research cruise GS16-204 are thanked, and especially Lisa Griem. The samples were provided through the ice2ice project, which was funded by the European Research Council under the European Community's Seventh Framework Programme (grant agreement 610055). This study is funded by the European Research Council under the European Union's Horizon 2020 research and innovation programme (grant agreement 818449, AGENSI).

REFERENCES

1. Griem, L., Voelker, A. H. L., Berben, S. M. P., Dokken, T. M. & Jansen, E. Insolation and Glacial Meltwater Influence on Sea-Ice and Circulation Variability in the Northeastern Labrador Sea During the Last Glacial Period. *Paleocean. and Paleoclimatol.* **34**, 1689–1709 (2019).
2. Scoto, F. *et al.* Sea ice fluctuations in the Baffin Bay and the Labrador Sea during glacial abrupt climate changes. *PNAS* **119**, e2203468119 (2022).
3. Pflaumann, U. *et al.* Glacial North Atlantic: Sea-surface conditions reconstructed by GLAMAP 2000. *Paleoceanography* **18**, 2002PA000774 (2003).
4. Jansen, E. *et al.* Past perspectives on the present era of abrupt Arctic climate change. *Nat. Clim. Chang.* **10**, 714–721 (2020).
5. Sadatzki, H. *et al.* Sea ice variability in the southern Norwegian Sea during glacial Dansgaard-Oeschger climate cycles. *Sci. Adv.* **5**, eaau6174 (2019).
6. Notz, D. & Stroeve, J. The Trajectory Towards a Seasonally Ice-Free Arctic Ocean. *Curr. Clim. Change Rep.* **4**, 407–416 (2018).
7. Stroeve, J. & Notz, D. Changing state of Arctic sea ice across all seasons. *Environ. Res. Lett.* **13**, 103001 (2018).
8. Wassmann, P., Duarte, C. M., Agustí, S. & Sejr, M. K. Footprints of climate change in the Arctic marine ecosystem. *Global Change Biol.* **17**, 1235–1249 (2011).
9. Brandt, S., Wassmann, P. & Piepenburg, D. Revisiting the footprints of climate change in Arctic marine food webs: An assessment of knowledge gained since 2010. *Front. Mar. Sci.* **10**, (2023).
10. Lannuzel, D. *et al.* The future of Arctic sea-ice biogeochemistry and ice-associated ecosystems. *Nat. Clim. Chang.* **10**, 983–992 (2020).
11. Flores, H. *et al.* Sea-ice decline could keep zooplankton deeper for longer. *Nat. Clim. Chang.* 1–9 (2023) doi:10.1038/s41558-023-01779-1.
12. Pedro, J. B. *et al.* Dansgaard-Oeschger and Heinrich event temperature anomalies in the North Atlantic set by sea ice, frontal position and thermocline structure. *Quatern. Sci. Rev.* **289**, 107599 (2022).
13. de Vernal, A., Gersonde, R., Goosse, H., Seidenkrantz, M.-S. & Wolff, E. W. Sea ice in the paleoclimate system: the challenge of reconstructing sea ice from proxies – an introduction. *Quatern. Sci. Rev.* **79**, 1–8 (2013).
14. Lejzerowicz, F. *et al.* Ancient DNA complements microfossil record in deep-sea subsurface sediments. *Biol. Lett.* **9**, 20130283 (2013).
15. De Schepper, S. *et al.* The potential of sedimentary ancient DNA for reconstructing past sea ice evolution. *ISME J* **13**, 2566–2577 (2019).

16. Armbrecht, L. *et al.* Ancient marine sediment DNA reveals diatom transition in Antarctica. *Nat. Commun.* **13**, 5787 (2022).
17. Zimmermann, H. H. *et al.* Marine ecosystem shifts with deglacial sea-ice loss inferred from ancient DNA shotgun sequencing. *Nat. Commun.* **14**, 1650 (2023).
18. Nguyen, N.-L. *et al.* Sedimentary ancient DNA: a new paleogenomic tool for reconstructing the history of marine ecosystems. *Front. Mar. Sci.* **10**, (2023).
19. You, D. *et al.* Last deglacial abrupt climate changes caused by meltwater pulses in the Labrador Sea. *Commun. Earth Environ.* **4**, 1–12 (2023).
20. Solignac, S., de Vernal, A. & Hillaire-Marcel, C. Holocene sea-surface conditions in the North Atlantic—contrasted trends and regimes in the western and eastern sectors (Labrador Sea vs. Iceland Basin). *Quatern. Sci. Rev.* **23**, 319–334 (2004).
21. Guillou, L. *et al.* The Protist Ribosomal Reference database (PR2): a catalog of unicellular eukaryote Small Sub-Unit rRNA sequences with curated taxonomy. *Nucleic Acids Res.* **41**, D597–D604 (2012).
22. Ludwig, J. A. & Cornelius, J. M. Locating Discontinuities along Ecological Gradients. *Ecology* **68**, 448–450 (1987).
23. Rahman, A. & de Vernal, A. Surface oceanographic changes in the eastern Labrador Sea: Nannofossil record of the last 31,000 years. *Mar. Geol.* **121**, 247–263 (1994).
24. Seidenkrantz, M.-S. *et al.* Evidence for influx of Atlantic water masses to the Labrador Sea during the Last Glacial Maximum. *Sci. Rep.* **11**, 6788 (2021).
25. Braeckman, U. *et al.* Carbon and nitrogen turnover in the Arctic deep sea: in situ benthic community response to diatom and coccolithophorid phytodetritus. *Biogeosci.* **15**, 6537–6557 (2018).
26. Cabrera-Brufau, M., Arin, L., Sala, M. M., Cermeño, P. & Marrasé, C. Diatom Dominance Enhances Resistance of Phytoplanktonic POM to Mesopelagic Microbial Decomposition. *Front. Mar. Sci.* **8**, (2021).
27. Fogwill, C. J. *et al.* Southern Ocean carbon sink enhanced by sea-ice feedbacks at the Antarctic Cold Reversal. *Nat. Geosci.* **13**, 489–497 (2020).
28. Steinsland, K. *et al.* Sea ice variability in the North Atlantic subpolar gyre throughout the Last Interglacial. *Quatern. Sci. Rev.* **313**, 108198 (2023).
29. Voelker, A. H. L. & Haflidason, H. Refining the Icelandic tephrachronology of the last glacial period – The deep-sea core PS2644 record from the southern Greenland Sea. *Global Planet. Change* **131**, 35–62 (2015).
30. Reimer, P. J. *et al.* IntCal13 and Marine13 Radiocarbon Age Calibration Curves 0–50,000 Years cal BP. *Radiocarbon* **55**, 1869–1887 (2013).
31. Ray, J. *et al.* Molecular gut content analysis demonstrates that Calanus grazing on Phaeocystis pouchetii and Skeletonema marinoi is sensitive to bloom phase but not prey density. *Mar. Ecol. Prog. Ser.* **542**, 63–77 (2016).
32. Martin, M. Cutadapt removes adapter sequences from high-throughput sequencing reads. *EMBnet.journal* **17**, 10–12 (2011).
33. Callahan, B. J. *et al.* DADA2: High-resolution sample inference from Illumina amplicon data. *Nat. Methods* **13**, 581–583 (2016).
34. Dufresne, Y., Lejzerowicz, F., Perret-Gentil, L. A., Pawlowski, J. & Cordier, T. SLIM: a flexible web application for the reproducible processing of environmental DNA metabarcoding data. *BMC Bioinformatics* **20**, 88 (2019).
35. Rognes, T., Flouri, T., Nichols, B., Quince, C. & Mahé, F. VSEARCH: a versatile open source tool for metagenomics. *PeerJ* **4**, e2584 (2016).

36. Davis, N. M., Proctor, D. M., Holmes, S. P., Relman, D. A. & Callahan, B. J. Simple statistical identification and removal of contaminant sequences in marker-gene and metagenomics data. *Microbiome* **6**, 226 (2018).
37. Oksanen, J. *et al.* vegan: Community Ecology Package. (2022).
38. Paulson, J. N., Stine, O. C., Bravo, H. C. & Pop, M. Differential abundance analysis for microbial marker-gene surveys. *Nat Methods* **10**, 1200–1202 (2013).
39. Salazar, G. EcolUtils: Utilities for community ecology analysis. (2023).

FIGURE CAPTIONS

Fig. 1. Location of the study site GS16-204-22 and other discussed sites in the Labrador Sea (1: HU90-13-13, 2: GS16-204-23CC, 3: MSM12/2-5-1).

Fig. 2. New metabarcoding data plotted against key paleoclimate and paleoceanographic records for the Last Glacial and Holocene. (a) NEEM oxygen isotope of water record, raw data (grey line) and 5-point running average (thick black line) (Gnikis et al. 2020). Greenland Stadials (GS) in black, Greenland Interstadials (GI) in red. (b) Planktic foraminifer *Neogloboquadrina pachyderma* (sinistral) oxygen isotopes of 22CC-A (black, Griem et al. 2019 and *this study*) and HU90-13-13 (blue, Hillaire-Marcel et al., 2003). Black triangles indicate ^{14}C AMS ages. (c) Relative abundance of dominant high-rank taxonomic groups in 22CC-B as determined by metabarcoding. (d) Normalised ASV richness, with 5-point running average (thick grey line). (e) Differentiation index of community composition, which shows statistically significant changes in composition as red dots. Background vertical shading is stadial-interstadial stratigraphy based on Rasmussen et al. (2014). All plots are on "thousand years before 2000 (ka b2k)", cf. Griem et al. (2019).

Fig. 3. New metabarcoding data plotted against key paleoclimate and paleoceanographic records for the period 40–36 ka b2k. (a) NEEM oxygen isotope of water record, raw data (grey line) and 5-point running average (thick black line) (Gnikis et al. 2020). (b) Planktic foraminifer *Neogloboquadrina pachyderma* (sinistral) oxygen isotopes of 22CC-A (Griem et al. 2019). Black triangles indicate ^{14}C AMS ages. (c) Relative abundance of *N. pachyderma* (sin) in 22CC-A (Griem et al. 2019). Values ≥98% indicate environments north of the Polar Front; values <94% indicate environments south of the Arctic Front. (d) Ice rafted detritus (IRD) records of 22CC-A (grey, Griem et al. 2019) and a targeted IRD record of 22CC-B (red, *this study*). (e) Sea ice biomarker record from GS16-204-23CC of Scoto et al. (2023). (f) Relative abundance of dominant high-rank taxonomic groups in 22CC-B as determined by metabarcoding. (g) Normalised ASV richness, with 5-point running average. (h) Differentiation index of community composition, which shows statistically significant changes in composition as red dots. Background vertical shading is stadial-interstadial stratigraphy based on Rasmussen et al. (2014). All plots are on "thousand years before 2000 (ka b2k)", cf. Griem et al. (2019).

Fig. 4. New metabarcoding data plotted against key paleoclimate and paleoceanographic records for the deglaciation and Holocene, 18–5 ka b2k. (a) NEEM oxygen isotope of water record, raw data (grey line) and 5-point running average (thick black line) (Gnikis et al. 2020). (b) Planktic foraminifer *Neogloboquadrina pachyderma* (sinistral) oxygen isotopes of 22CC-A (black, Griem et al. 2019 and *this study*) and HU90-13-13 (blue, Hillaire-Marcel et al., 2003). Black triangles indicate ^{14}C AMS ages. (c) Sea ice biomarker IP_{25} and its accumulation rates (shading) from MSM12/2-5-1 (You et al. 2023). (d) Sea ice biomarker HBI-III(Z) and its accumulation rates (shading) from MSM12/2-5-1 (You et al. 2023). (e) Alkenone sea surface temperatures from MSM12/2-5-1 (You et al. 2023) using the Filippova et al. (2016) calibration. (f) Relative abundance of dominant high-rank taxonomic groups in 22CC-B as determined by metabarcoding. (h) Differentiation index, which shows statistically significant changes in diversity as red dots. Background vertical blue shading are meltwater pulses (1-4) from You et al. (2023. Black vertical lines show the boundaries of the Younger Dryas (YD), the Bølling-Allerød (B-A) and Heinrich Stadial 1. All plots are on "thousand years before 2000 (ka b2k)", cf. Griem et al. (2019).

SUPPLEMENTARY FIGURES AND TABLES

Supplementary Fig. S1. a) Colour reflectance data plotted against core depth for cores 22CC-A and 22CC-B. b) Colour reflectance data for cores 22CC-A and 22CC-B, plotted on the 22CC-B core depth. Correlations from 22CC-A to 22CC-B were made based on a* parameter (r=0.834); L* and b* are shown as support for the a* correlation.

Supplementary Fig. S2. Age depth plot for 22CCB shows a higher sedimentation rate in the Holocene compared to the last glacial. Ages from 22CC-A were transferred to 22CC-B via the a* parameter correlation (see Suppl. Fig. S1).

Supplementary Table S1. Lightness parameters (L8, a*, b*) raw data, including tie points between 22CC-A and 22CC-B based on the correlation of the a* parameter of both holes.

Supplementary Table S2. Age model raw data.

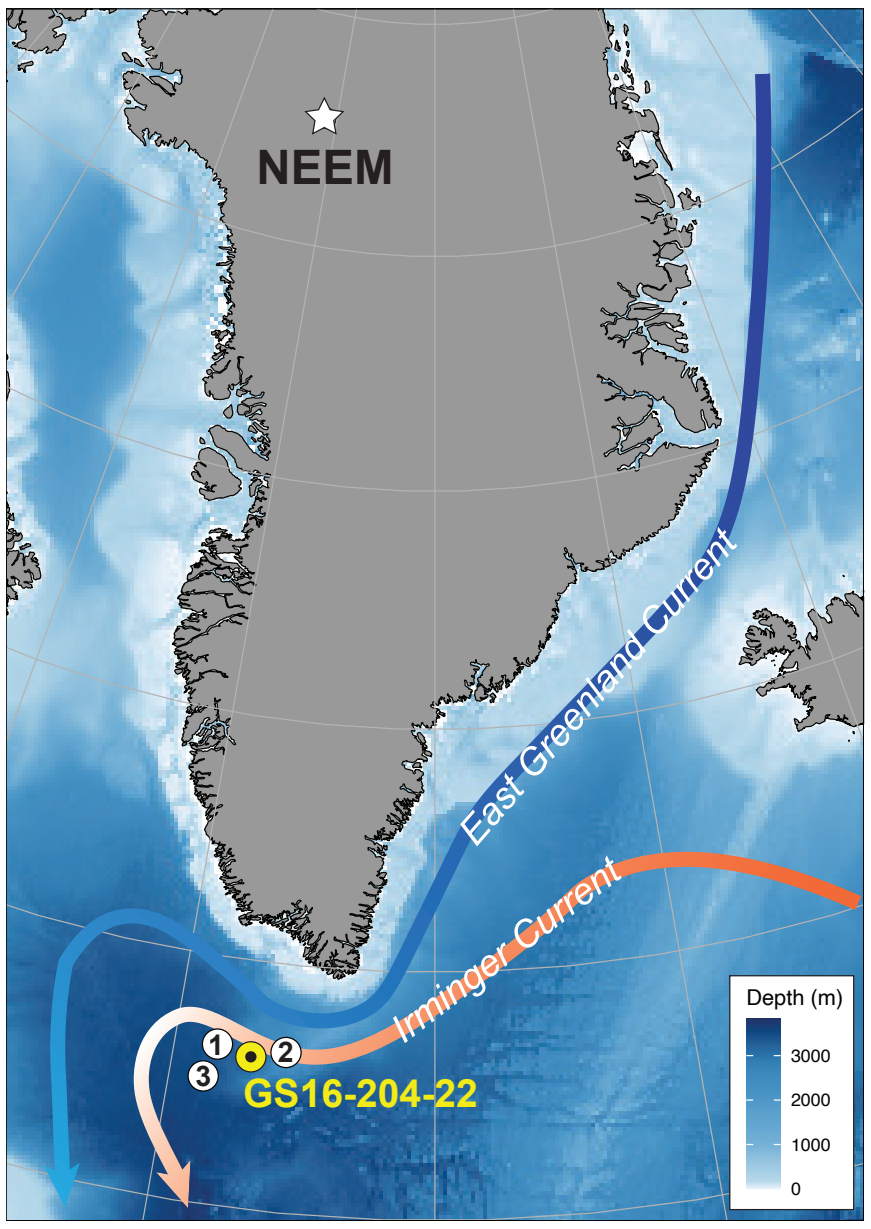
NEEM
East Greenland Current
Irminger Current
1
2
3
GS16-204-22
Depth (m)
3000
2000
1000
0

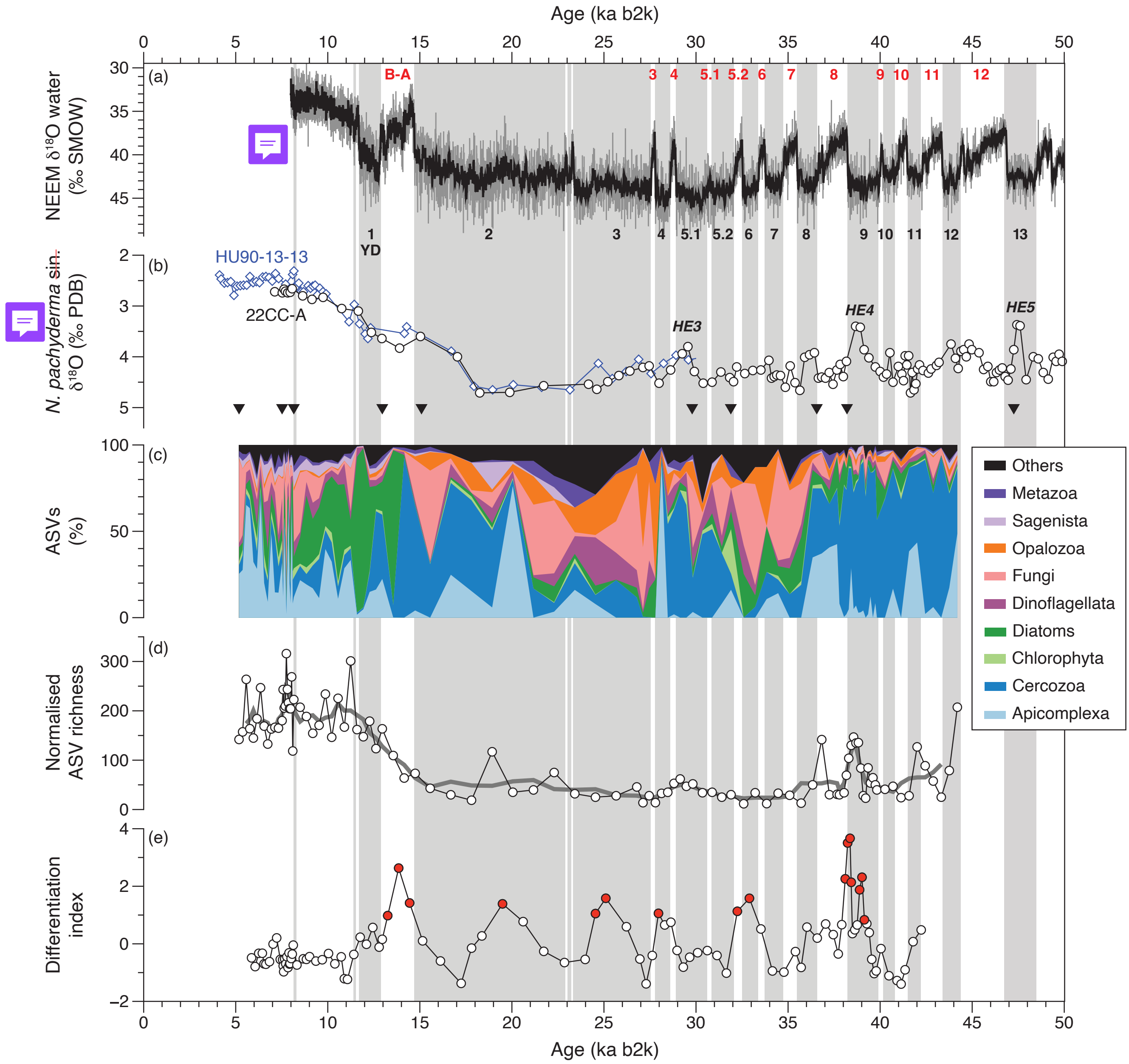

Age (ka b2k)
NEEM δ18O water (‰ SMOW)
B-A
3 4 5.1 5.2 6 7 8 9 10 11 12
1 YD 2 3 4 5.1 5.2 6 7 8 9 10 11 12 13
HU90-13-13
22CC-A
N. pachyderma sin. δ18O (‰ PDB)
HE3
HE4
HE5
ASVs (%)
Others
Metazoa
Sagenista
Opalozoa
Fungi
Dinoflagellata
Diatoms
Chlorophyta
Cercozoa
Apicomplexa
Normalised ASV richness
Differentiation index
(a) (b) (c) (d) (e)

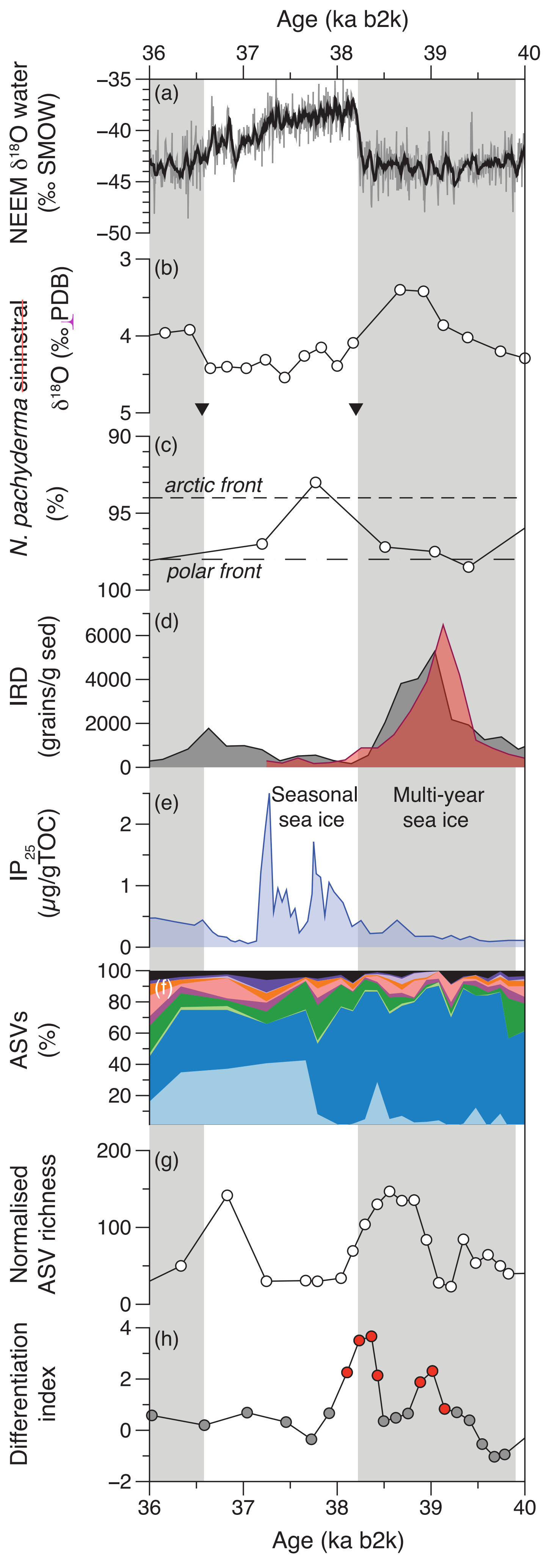

Age (ka b2k)
36
37
38
39
40
NEEM δ18O water (‰ SMOW)
−35
−40
−45
−50
(a)
3
4
5
(b)
δ18O (‰ PDB)
N. pachyderma sininstral
(%)
90
95
100
(c)
arctic front
polar front
IRD (grains/g sed)
6000
4000
2000
0
(d)
IP25 (μg/gTOC)
2
1
0
(e)
Seasonal sea ice
Multi-year sea ice
ASVs (%)
100
80
60
40
20
(f)
Normalised ASV richness
200
100
0
(g)
Differentiation index
4
2
0
−2
(h)
36
37
38
39
40
Age (ka b2k)

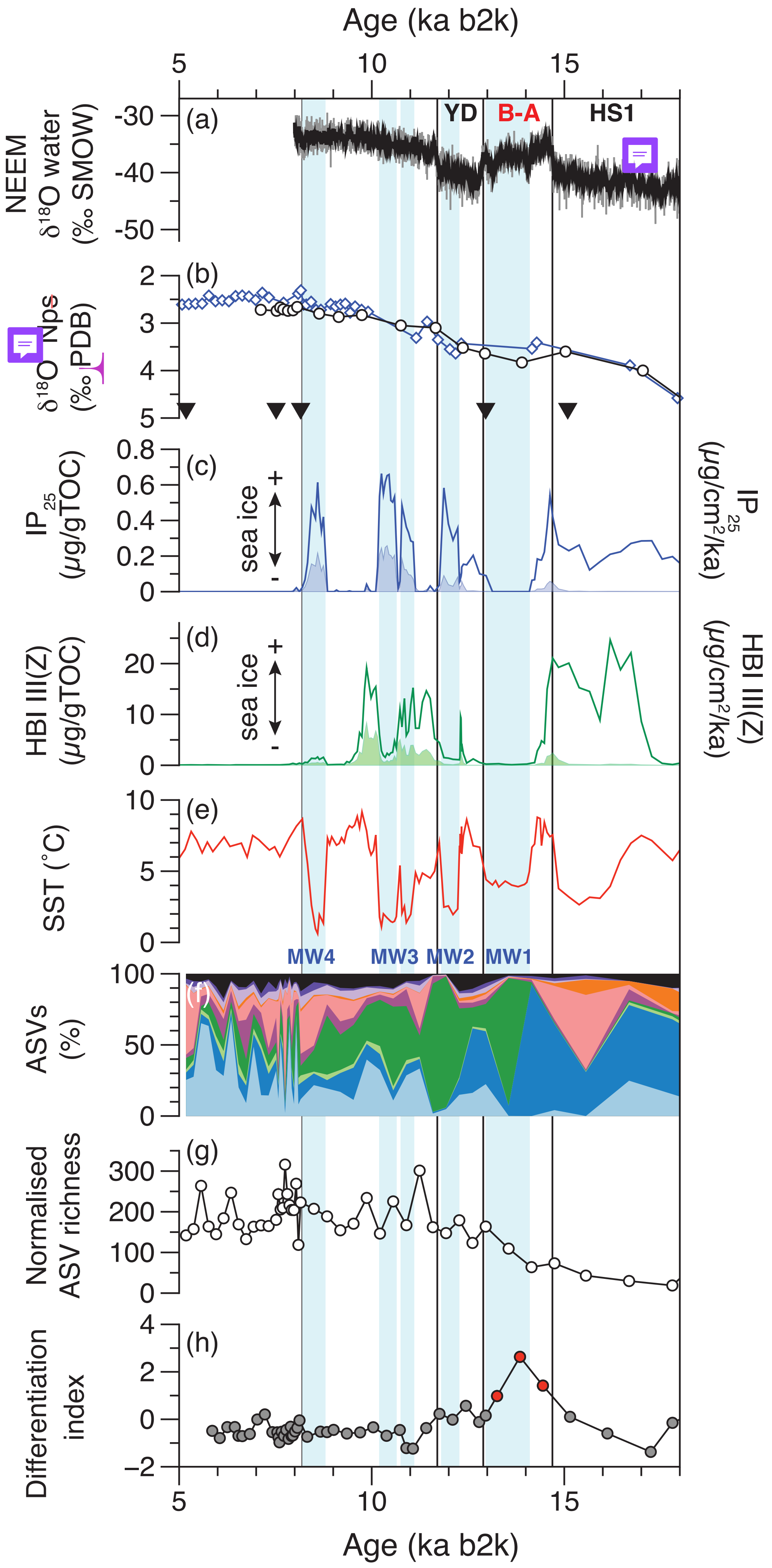

Age (ka b2k)
NEEM δ18O water (‰ SMOW)
δ18O Nps (‰ PDB)
IP25 (μg/gTOC)
IP25 (μg/cm2/ka)
HBI III(Z) (μg/gTOC)
HBI III(Z) (μg/cm2/ka)
sea ice
SST (°C)
ASVs (%)
Normalised ASV richness
Differentiation index
YD
B-A
HS1
MW4
MW3
MW2
MW1
(a)
(b)
(c)
(d)
(e)
(f)
(g)
(h)
