# Supplementary material for: Changing sea ice cover led to marine biodiversity shifts in the Late Quaternary
Source: Commun Earth Environ. 2026 Jun 4;7(1):732. doi: 10.1038/s43247-026-03696-5 (PMC13549946; doi:10.1038/s43247-026-03696-5)
Supplement: Supplementary file 2 [file 43247_2026_3696_MOESM2_ESM.pdf]

Supplementary Figure S1. Correlation of GS16-204-CC-A with GS16-204-CC-B using sediment colour data.
a) Colour reflectance data plotted against core depth for cores 22CC-A and 22CC-B.
b) Colour reflectance data for cores 22CC-A and 22CC-B, plotted on the 22CC-B core depth. Correlations from 22CC-A to 22CC-B were made based on a* parameter (r=0.834); L* and b* are shown as support for the a* correlation.

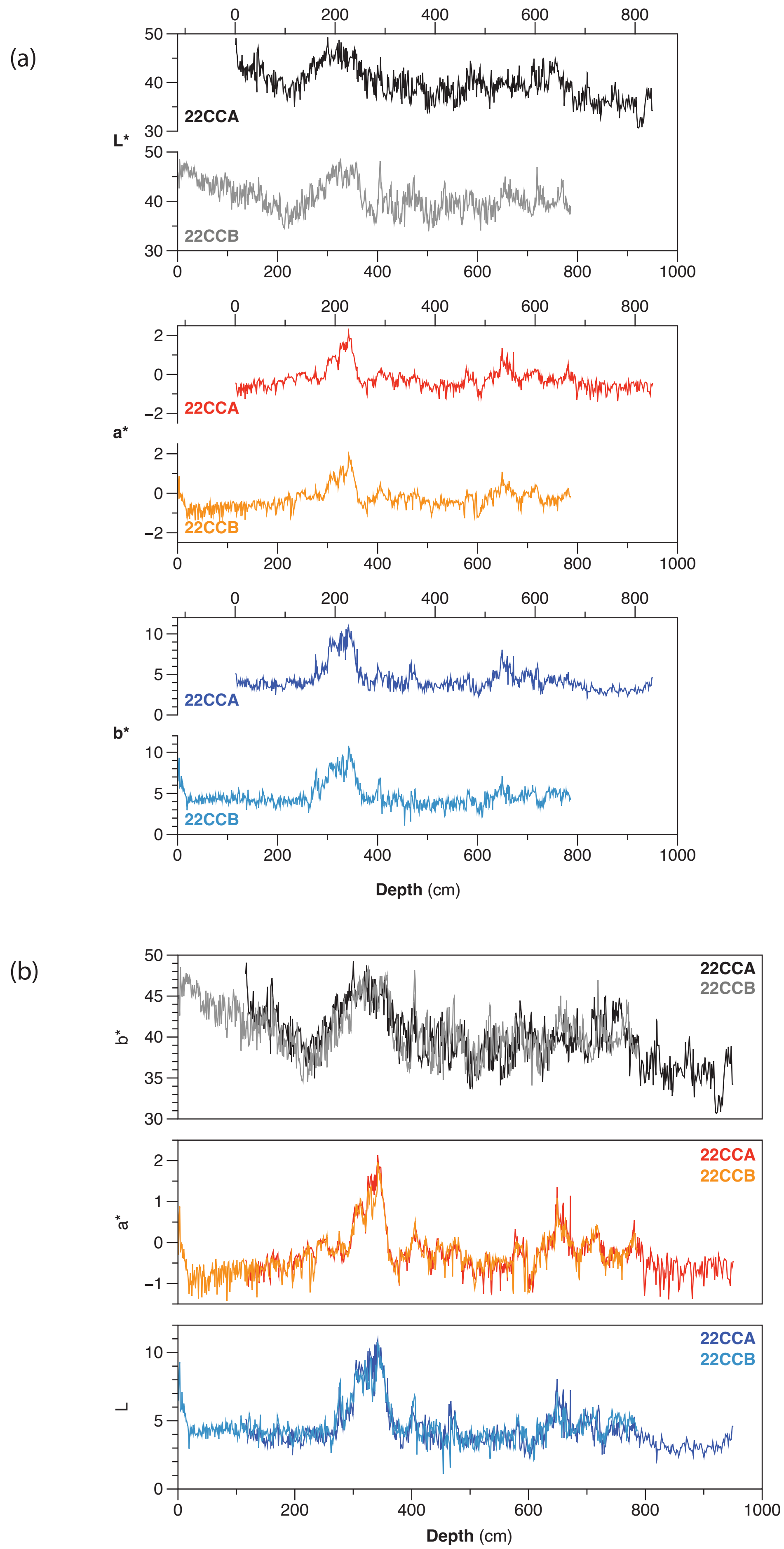

Supplementary Figure S2. Age depth plot for 22CC-B shows a higher sedimentation rate in the Holocene compared to the last glacial. Ages from 22CC-A were transferred to 22CC-B via the a* parameter correlation (see Suppl. Fig. S1).

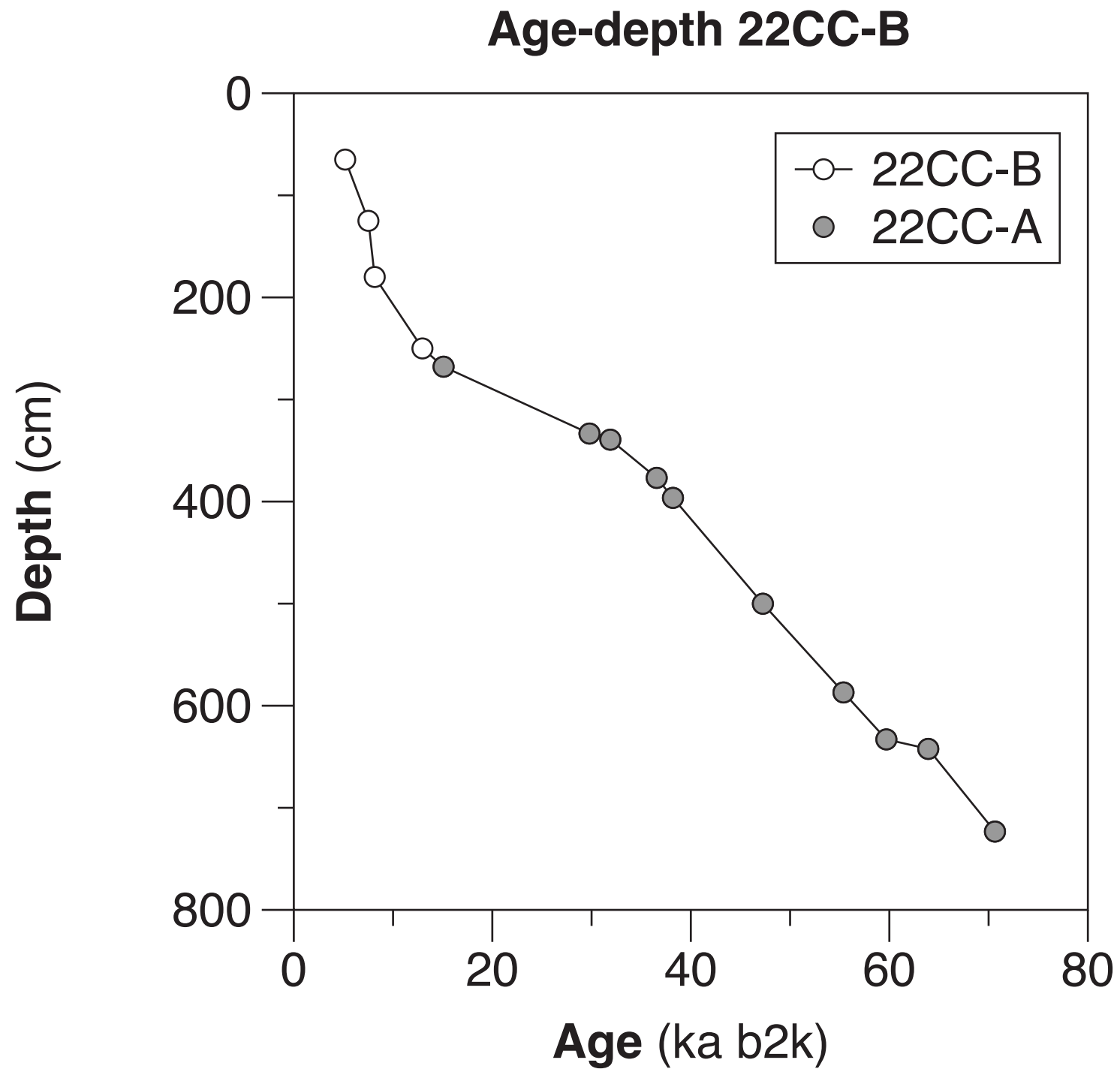

Supplementary Table S1. Tiepoints between 22CC-A and 22CC-B based on the correlation of the a* parameter of both holes.

GS16-204-22CC-A Depth (cm)		GS16-204-22CC-B Depth (cm)
2	ties to	117
10	ties to	126
12	ties to	127
21	ties to	135
28	ties to	139
39	ties to	150
41	ties to	153
46	ties to	158
48	ties to	159
50	ties to	163
54	ties to	166
57	ties to	171
62	ties to	173
65	ties to	178
68	ties to	181
70	ties to	187
79	ties to	194
84	ties to	196
87	ties to	198
94	ties to	210
113	ties to	235
124	ties to	245
138	ties to	256
159	ties to	276
164	ties to	279
172	ties to	283
195	ties to	301
210	ties to	321
213	ties to	324
218	ties to	331
234	ties to	342
242	ties to	356
249	ties to	360
268	ties to	380
277	ties to	391
282	ties to	396
289	ties to	406
291	ties to	408
295	ties to	416
304	ties to	425
307	ties to	430
313	ties to	433
329	ties to	442

GS16-204-22CC-A Depth (cm)		GS16-204-22CC-B Depth (cm)
332	ties to	447
344	ties to	457
347	ties to	463
360	ties to	474
361	ties to	477
363	ties to	480
366	ties to	484
385	ties to	501
395	ties to	510
404	ties to	523
419	ties to	536
436	ties to	552
448	ties to	564
464	ties to	575
476	ties to	589
480	ties to	597
496	ties to	613
512	ties to	631
530	ties to	647
532	ties to	649
538	ties to	657
543	ties to	663
565	ties to	682
569	ties to	687
603	ties to	722
615	ties to	732
618	ties to	738
636	ties to	753
642	ties to	760
650	ties to	772
668	ties to	783
672	ties to	787
677	ties to	790
680	ties to	795
684	ties to	802
693	ties to	809
701	ties to	818
706	ties to	821
710	ties to	830
713	ties to	835
717	ties to	840
724	ties to	847
730	ties to	857

Supplementary Table S2. Age model for 22CC-A (Griem et al. 2019) and 22CC-B (this study).

22CC-A *			22CC-B_cm		
**Depth (cm)**	**Age (ka b2k)**	**Tie points**	**Depth (cm)**	**Age (ka b2k)**	**Tie points**
			65.0	5.180	AMS 14C
			125.0	7.518	AMS 14C
			180.0	8.155	AMS 14C
			250.0	12.960	AMS 14C
150.5	15.090	AMS 14C	267.9	15.090	transferred from 22CC-A
221.5	29.790	d18O	333.4	29.790	transferred from 22CC-A
230.3	31.890	AMS 14C	339.4	31.890	transferred from 22CC-A
265.0	36.560	d18O	376.8	36.560	transferred from 22CC-A
282.3	38.200	d18O	396.4	38.200	transferred from 22CC-A
384.0	47.260	d18O	500.1	47.260	transferred from 22CC-A
474.3	55.380	ANAAZ II ash layer	587.0	55.380	transferred from 22CC-A
514.3	59.700	d13C	633.0	59.700	transferred from 22CC-A
524.8	63.920	d18O	642.3	63.920	transferred from 22CC-A
604.5	70.640	d13C	723.3	70.640	transferred from 22CC-A

* Age control points for sediment core GS16-204-22CC-A from Griem et al., 2019 (table 2)

Age control points from 22CC-A were transferred to 22CC-B via the correlation of the a* parameter (see Table S1

All 14C ages were calibrated with Marine13 and no reservoir correction, and added 50 years to get to b2k ages
