# Supplementary material for: Changing sea ice cover led to marine biodiversity shifts in the Late Quaternary
Source: Commun Earth Environ. 2026 Jun 4;7(1):732. doi: 10.1038/s43247-026-03696-5 (PMC13549946; doi:10.1038/s43247-026-03696-5)
Supplement: Supplementary file 3 [file 43247_2026_3696_MOESM3_ESM.pdf]

Please wait...

If this message is not eventually replaced by the proper contents of the document, your PDF viewer may not be able to display this type of document.

You can upgrade to the latest version of Adobe Reader for Windows®, Mac, or Linux® by visiting http://www.adobe.com/go/reader_download.

For more assistance with Adobe Reader visit http://www.adobe.com/go/acrreader.

Windows is either a registered trademark or a trademark of Microsoft Corporation in the United States and/or other countries. Mac is a trademark of Apple Inc., registered in the United States and other countries. Linux is the registered trademark of Linus Torvalds in the U.S. and other countries.
